# Modeling diabetic endothelial dysfunction with patient‐specific induced pluripotent stem cells

**DOI:** 10.1002/btm2.10592

**Published:** 2023-08-30

**Authors:** Rayyan Gorashi, Nancy Rivera‐Bolanos, Caitlyn Dang, Cedric Chai, Beatrix Kovacs, Sara Alharbi, Syeda Subia Ahmed, Yogesh Goyal, Guillermo Ameer, Bin Jiang

**Affiliations:** ^1^ Department of Biomedical Engineering Northwestern University Evanston and Chicago Illinois USA; ^2^ Center for Advanced Regenerative Engineering Northwestern University Evanston Illinois USA; ^3^ Department of Surgery Feinberg School of Medicine, Northwestern University Chicago Illinois USA; ^4^ Department of Cell and Developmental Biology Northwestern University Feinberg School of Medicine Chicago Illinois USA; ^5^ Center for Synthetic Biology Northwestern University Chicago Illinois USA; ^6^ Center for Reproductive Science Northwestern University Chicago Illinois USA; ^7^ Robert H. Lurie Comprehensive Cancer Center Northwestern University Feinberg School of Medicine Chicago Illinois USA

**Keywords:** cardiovascular diseases, diabetes mellitus, disease modeling, drug screening, endothelial cells, induced pluripotent stem cells

## Abstract

Diabetes is a known risk factor for various cardiovascular complications, mediated by endothelial dysfunction. Despite the high prevalence of this metabolic disorder, there is a lack of in vitro models that recapitulate the complexity of genetic and environmental factors associated with diabetic endothelial dysfunction. Here, we utilized human induced pluripotent stem cell (iPSC)‐derived endothelial cells (ECs) to develop in vitro models of diabetic endothelial dysfunction. We found that the diabetic phenotype was recapitulated in diabetic patient‐derived iPSC‐ECs, even in the absence of a diabetogenic environment. Subsequent exposure to culture conditions that mimic the diabetic clinical chemistry induced a diabetic phenotype in healthy iPSC‐ECs but did not affect the already dysfunctional diabetic iPSC‐ECs. RNA‐seq analysis revealed extensive transcriptome‐wide differences between cells derived from healthy individuals and diabetic patients. The in vitro disease models were used as a screening platform which identified angiotensin receptor blockers (ARBs) that improved endothelial function in vitro for each patient. In summary, we present in vitro models of diabetic endothelial dysfunction using iPSC technology, taking into account the complexity of genetic and environmental factors in the metabolic disorder. Our study provides novel insights into the pathophysiology of diabetic endothelial dysfunction and highlights the potential of iPSC‐based models for drug discovery and personalized medicine.

**Translational Impact Statement**

This study establishes a robust disease model for diabetic vascular complications using patient‐derived induced pluripotent stem cells, with significant differences between cells from healthy individuals and diabetic patients. Drug screening using this disease model identifies potential therapeutics that could improve endothelial function, which has significant translational and clinical significance for personalized medicine in the management of diabetic vascular complications. This work bridges the gap between preclinical animal studies and human clinical trials, potentially improving patient outcomes and guiding the clinical management of diabetic vascular complications.

## 1 INTRODUCTION

Diabetes mellitus is a growing public health problem with known contributions to lifestyle and genetics.^1^ In the United States alone, its prevalence has reached about 34 million people as of 2020, with another 88 million people diagnosed as prediabetic.^2^ The recent COVID‐19 pandemic has posed a substantial risk for those living with diabetes, resulting in significantly increased disease severity and higher mortality after the coronavirus infection.^3^ Diabetes mellitus is associated with increased risks of cardiovascular diseases and vasculopathy, including macrovascular (e.g., coronary and peripheral artery disease) and microvascular complications (e.g., diabetic foot ulcer and retinopathy).^4^ Despite significant recent advances in blood glucose monitoring and glycemic control, debilitating vascular complications remain in most diabetic patients.^5^ Chronic exposure to hyperglycemia, hyperlipidemia, and oxidative stress presented in diabetes is believed to cause vascular damage that is persistent even after glucose normalization.^6^ Unfortunately, no specific clinical treatment currently exists to improve these cardiovascular outcomes. There is, thus, an urgent need to develop effective therapeutics to restore vascular functions for those living with diabetes.

Although the detailed mechanisms of the pathogenesis of diabetic vascular diseases are still not completely understood, all mechanisms converge on the endothelium as an important disease target.^7^, ^8^ The vascular endothelium serves several functions for the maintenance of vascular tone, mechanotransduction, injury repair, metabolism, barrier integrity, metabolism, angiogenesis, and hemostasis.^9^, ^10^ Endothelial dysfunction—characterized by a reduction of nitric oxide bioavailability, an increase in reactive oxygen species (ROS) and a pro‐inflammatory, pro‐thrombotic milieu—is a key marker in all forms of diabetic vascular complications.^11^, ^12^ To date, a number of preclinical animal models have been developed for diabetes and diabetic vascular complications.^13^ However, animal models do not always faithfully recapitulate the human condition, which has contributed to the lack of clinical translational success in this area.^14^ Development of human disease models that are representative of diabetic endothelial dysfunction in vitro will complement the current preclinical animal models and facilitate therapeutic discovery to restore the endothelial function in diabetes.

The advent of induced pluripotent stem cell (iPSC) technology offers an exceptional opportunity for creating disease‐ and patient‐specific cellular models, investigating underlying mechanisms, and optimizing therapy.^15^ Our study aims to develop and validate in vitro models of diabetic endothelial dysfunction using iPSC‐derived endothelial cells (iPSC‐ECs) from diabetic and healthy individuals to dissect the underlying mechanisms and identify new therapeutic targets. Using iPSC technology, we generated patient‐specific ECs from healthy subjects and diabetic patients, evaluated their baseline endothelial functions, and identified molecular signatures of endothelial dysfunction in diabetes with transcriptome analysis. Furthermore, we investigated the role of a diabetogenic treatment in inducing the diabetic phenotype in healthy and diabetic ECs. Lastly, using the patient‐derived iPSC‐ECs as in vitro disease models, we performed drug screening and identified angiotensin receptor blockers (ARBs) that improved endothelial function in vitro for each patient. The findings from our study may provide new insights into the pathophysiology of diabetic endothelial dysfunction and contribute to the development of personalized therapies for diabetic cardiovascular complications.

## 2 RESULTS

### 2.1 ECs could be generated from diabetic patients and healthy subjects with iPSC technology

**FIGURE 1:**
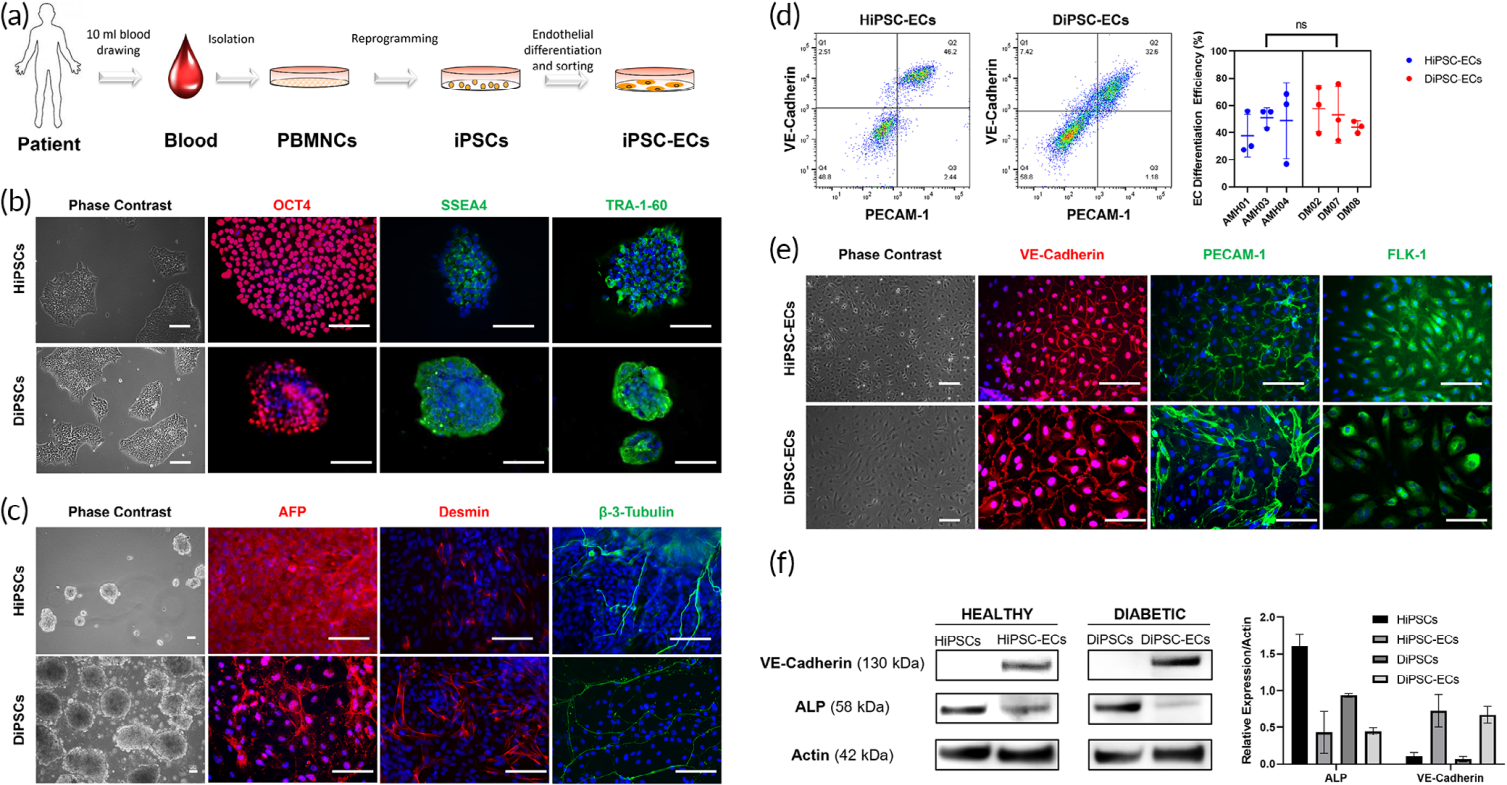
Generation and characterization of iPSCs and iPSC‐ECs from healthy subjects and diabetic patients. (a) Schematic representation of the general process of iPSC‐EC generation from the patient blood samples using iPSC technology; (b) Representative images of iPSC colonies derived from three age‐matched healthy control subjects (HiPSCs) and three diabetic patients (DiPSCs) expressing pluripotency markers OCT4, SSEA4, and TRA‐1‐60; (c) Representative images of embryoid bodies formed from HiPSCs and DiPSCs in vitro expressing markers of the three germ layers: Alpha Fetoprotein (endoderm), Desmin (mesoderm) and β‐3‐Tubulin (ectoderm); (d) Flow cytometry analysis after EC differentiation from HiPSCs and DiPSCs for EC markers VE‐Cadherin and PECAM‐1 prior to cell soring with no statistically significant difference in EC differentiation efficiency; (e) MACS sorted HiPSC‐ECs and DiPSC‐ECs exhibiting cobblestone morphology and expressing endothelial markers VE‐Cadherin, PECAM‐1 and Flk‐1; (f) Representative western blots and quantitative analysis of iPSCs (healthy and diabetic) expressing alkaline phosphatase (ALP) and iPSC‐ECs (healthy and diabetic) expressing VE‐Cadherin. Scale bar = 100 μm.

To generate patient‐specific ECs, we reprogrammed the peripheral blood mononuclear cells (PBMCs) from three diabetic patients and three age‐matched healthy subjects (Table 1) into iPSCs, followed by directed differentiation into ECs under xeno‐free conditions (Figure 1a). All human subject studies have been approved by the Northwestern University Internal Review Board (IRB), and written informed consents were obtained from each participant prior to the blood collection. iPSC colonies generated from healthy subjects (HiPSCs) and diabetic patients (DiPSCs) with Sendai virus encoding OCT4, SOX2, KLF4, and c‐MYC were morphologically and phenotypically similar to each other, all expressing pluripotency markers OCT4, SSEA4 and TRA‐1‐60 (Figure 1b). All iPSC lines exhibited normal human female (AMH 01, AMH 03, and DM 02) and male (AMH 04, DM 07, and DM 08) karyotype up to passage 15 (Figure S1). DNA fingerprinting verified that each patient‐specific iPSC line was derived from each individual parental PBMC line without cross‐contamination (Table S1). All iPSC lines were able to form embryoid bodies in vitro, and the differentiated cells expressed markers for the endoderm (Alpha Fetoprotein), mesoderm (Desmin), and ectoderm (ß‐3‐Tubulin). (Figure 1c) There was no characteristic difference observed between the HiPSCs and DiPSCs.

**TABLE 1:** Human subject information.

Group	Code	Age	Sex	Race	Cardiovascular complications
Healthy	AMH 01	62	Female	Caucasian	N/A
	AMH 03	63	Female	Caucasian	N/A
	AMH 04	53	Male	Black	N/A
Diabetic	DM 02	74	Female	Caucasian	Peripheral arterial disease, coronary artery disease
	DM 07	68	Male	Caucasian	Peripheral arterial disease
	DM 08	56	Male	Black	Peripheral arterial disease, coronary artery disease

ECs were differentiated from HiPSCs and DiPSCs following a previously published protocol,^16^ which resulted in similar differentiation efficiencies (HiPSC‐ECs: 46.4% ± 7.8%; DiPSC‐ECs: 51.6 ± 6.9%, *p* = 0.4307), assessed via flow cytometry for VE‐Cadherin^+^ and PECAM‐1^+^ cells (Figure 1d). VE‐Cadherin^+^ cells were isolated via magnetic‐assisted cell sorting (MACS) and cultured in EC medium for subsequent experiments. All iPSC‐ECs exhibited typical endothelial cobblestone morphology and expressed endothelial markers VE‐Cadherin, PECAM‐1, and Flk‐1 (Figure 1e). Western blotting verified the expression of the endothelial marker VE‐Cadherin in both HiPSC‐ECs and DiPSC‐ECs, as well as the expression of the pluripotency marker ALP in both HiPSCs and DiPSCs (Figure 1f). DNA fingerprinting confirmed that each patient‐specific iPSC‐EC line was derived from the same parental PBMC and iPSC lines (Table S1).

### 2.2 DiPSC‐ECs exhibit baseline endothelial dysfunction in the absence of a diabetogenic microenvironment

**FIGURE 2:**
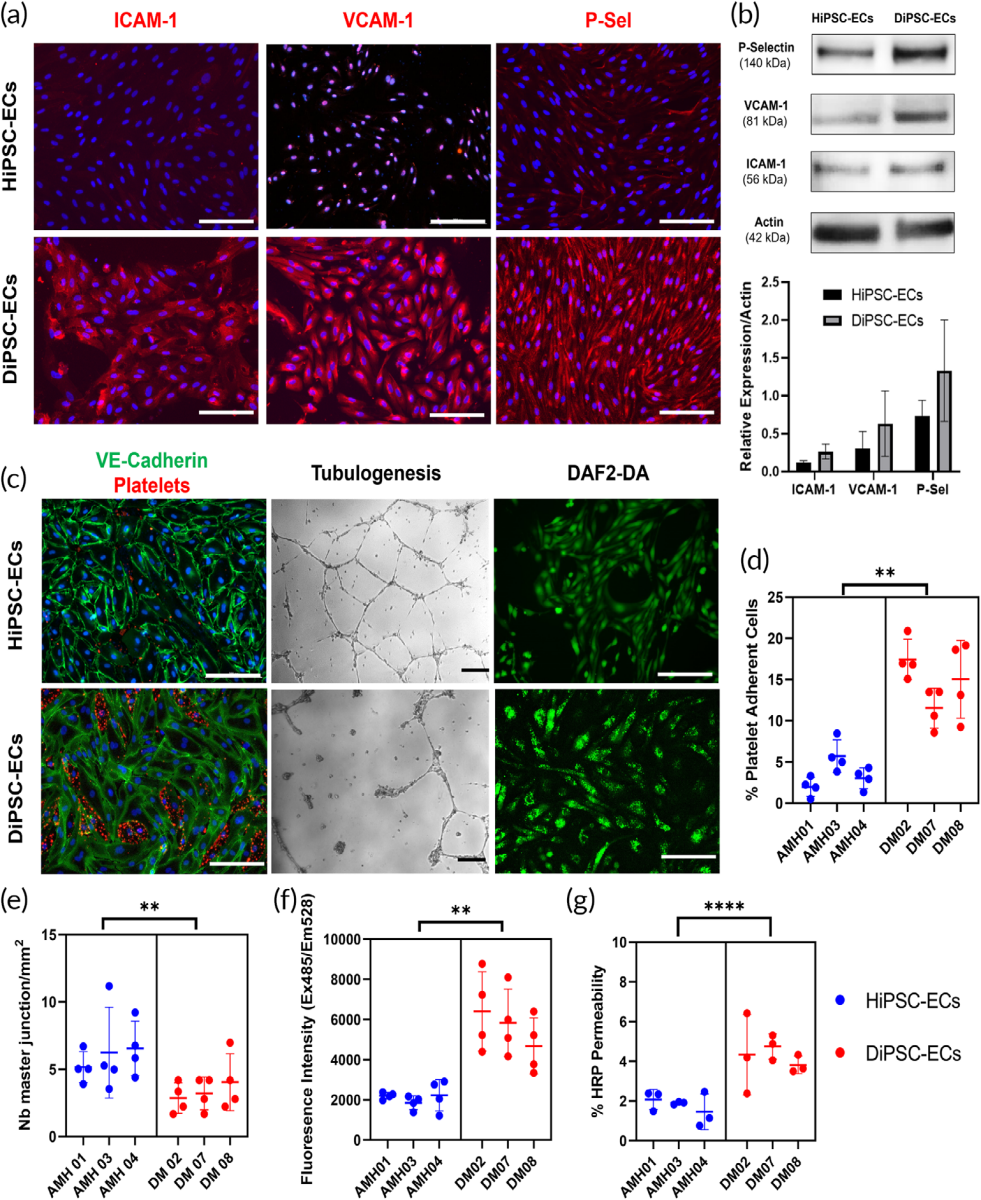
Functional assessment of HiPSC‐ECs and DiPSC‐ECs at the baseline level. (a) Representative images of HiPSC‐ECs staining negative for adhesion molecules (ICAM‐1, VCAM‐1 and P‐Selectin) while DiPSC‐ECs staining positive for the adhesion molecules; (b) Representative western blots and quantitative analysis of DiPSC‐ECs expressing elevated levels of ICAM‐1, VCAM‐1 and P‐Selectin relative to HiPSC‐ECs; (c) Representative images of HiPSC‐ECs with low platelet adhesion, organized tube formation, and intracellular nitric oxide production, whereas DiPSC‐ECs exhibiting high platelet binding, disorganized tubulogenesis, but still with intracellular nitric oxide production; (d) Quantification of platelet adherent cells indicating DiPSC‐ECs exhibit higher thrombogenicity than HiPSC‐ECs; (e) Quantification of master junction density in tubulogenesis analysis indicating DiPSC‐ECs exhibit lower angiogenic potential than HiPSC‐ECs; (f) Quantification of intracellular ROS production with DCF‐DA assay indicating DiPSC‐ECs exhibit higher oxidative stress than HiPSC‐ECs; (g) Quantification of permeability by an HRP diffusion assay indicating DiPSC‐ECs have higher permeability than HiPSC‐ECs thus impaired barrier function. Scale bar = 200 μm. *p < 0.05. **p < 0.01. n.s. not significant.

We next evaluated endothelial functions for HiPSC‐ECs and DiPSC‐ECs at the baseline level under normal EC culture conditions at early passages (passages 2–3). DiPSC‐ECs from all diabetic patients in the study expressed inflammation‐related markers, including VCAM‐1, ICAM‐1, and P‐Selectin, in contrast to HiPSC‐ECs from healthy subjects, which did not express those proteins (Figure 2a). Western blotting study verified the elevated expression of VCAM‐1, ICAM‐1, and P‐Selectin in DiPSC‐ECs relative to HiPSC‐ECs (Figure 2b). Interaction of HiPSC‐EC monolayers with healthy human platelets exhibited limited platelet adhesion, indicating functional anti‐thrombogenic activity of the HiPSC‐ECs (Figure 2c). In contrast, platelets aggregated on a subpopulation of DiPSC‐ECs from diabetic patients, suggesting compromised thrombo‐resistant functions of the cells. A quantitative analysis revealed a significantly higher percentage of platelet‐adherent cells from DiPSC‐ECs than HiPSC‐ECs (*p* = 0.0056) (Figure 2d). A tubulogenesis assay with HiPSC‐ECs seeded onto a basement membrane hydrogel (Matrigel™) formed interconnected tubular networks (Figure 2c), indicating functional angiogenetic potential. However, DiPSC‐ECs seeded onto Matrigel™ formed disorganized networks with a significantly lower number of master junctions, when compared with HiPSC‐ECs (*p* = 0.0028) (Figure 2c,e). DiPSC‐ECs also exhibited significantly higher amount of intracellular ROS when compared to HiPSC‐ECs (*p* = 0.0024) (Figure 2f). However, no significant difference was detected in nitric oxide production with an intracellular DAF2‐DA assay (Figure 2c) or an extracellular nitrite assay (*p* = 0.6056). Lastly, monolayers formed with DiPSC‐ECs exhibited disrupted barrier function and thus increased permeability, which allowed for a significantly higher amount of HRP diffusion, when compared with HiPSC‐EC monolayer (*p* < 0.0001) (Figure 2g). All experiments were performed under a normal endothelial culture condition in the absence of a diabetic‐like culture condition.

### 2.3 Transcriptome‐wide differences exist between cells from healthy and diabetic patients

**FIGURE 3:**
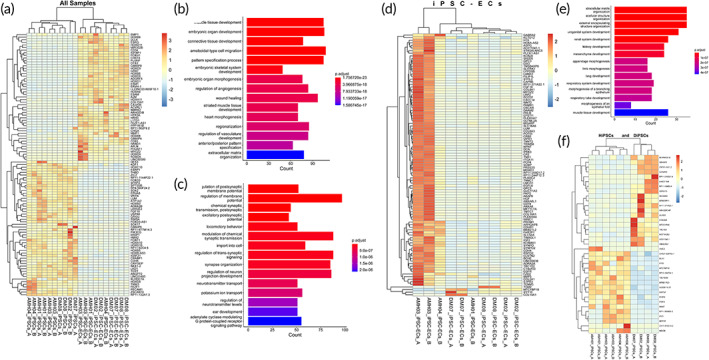
RNA‐seq differential gene expression analysis comparing iPSCs and iPSC‐ECs between healthy and diabetic groups. (a) Heatmap of the 100 most up‐ and downregulated genes comparing iPSCs and iPSC‐ECs; (b,c) Gene ontology (GO) analysis of both upregulated and downregulated genes between iPSCs and iPSC‐ECs; (d) Heatmap of the 37 most up‐ and downregulated genes comparing HiPSC‐ECs and DiPSC‐ECs; (e) Joint GO analysis of both upregulated and downregulated genes between HiPSC‐ECs and DiPSC‐ECs; (f) Heatmap of the 37 most up‐ and downregulated genes comparing HiPSCs and DiPSCs.

We wondered if there were any global transcriptional differences between cells isolated from healthy individuals and diabetic patients. To answer this, we isolated RNA and performed RNA sequencing of iPSCs and Ipsc‐ECs from the two groups (total 24 samples; see Methods 4.8) for identifying transcriptome‐wide differences. We first asked whether we see differences between the iPSCs and iPSC‐ECs differentiated populations for both healthy and disease patients. Indeed, with both principal component analysis (PCA) (Figure S2) and a conservative filtering of differential expressed genes (log2FC > 2 and *p*_adj_ < 0.01), we found a total of 3464 genes being differentially expressed (Figure 3a; Figure S3; Table S2). Importantly, as expected, our Gene Ontology (GO) analysis^17^ revealed that these genes upregulated in differentiated samples corresponded to angiogenesis, vascular development, and muscle tissue development (Figure 3b,c). This result establishes the power and validity of our bulk sequencing approach. We next asked what kind of transcriptome‐wide differences exist between cells from healthy individuals and diabetic patients. We performed differential expression analysis separately for iPSCs and iPSC‐ECs and found significant differences between cells from healthy individuals vs. diabetic patients (Figure 3d,e; Figure S4; Table S3). For example, with a log2FC > 2 and *p*_adj_ < 0.01, we found 245 (and 378 for a log2FC > 1) total genes differentially expressed between HiPSC‐ECs and DiPSC‐ECs (Figure 3d). Our GO analysis revealed that consistent with our protein‐level analysis, these genes corresponded to extracellular matrix structure and organization, among others (Figure 3e). Similarly, we also found differences, albeit to a lesser degree (total 37 different genes log2FC > 2 and *p*_adj_ < 0.01), between HiPSCs and DiPSCs (Figure 3f; Figure S5; Table S4). Together, our experiments reveal extensive transcriptome‐wide differences between cells from healthy and diabetic patients.

### 2.4 Exposure to a diabetogenic environment induces endothelial dysfunction in HiPSC‐ECs without affecting DiPSC‐ECs

**FIGURE 4:**
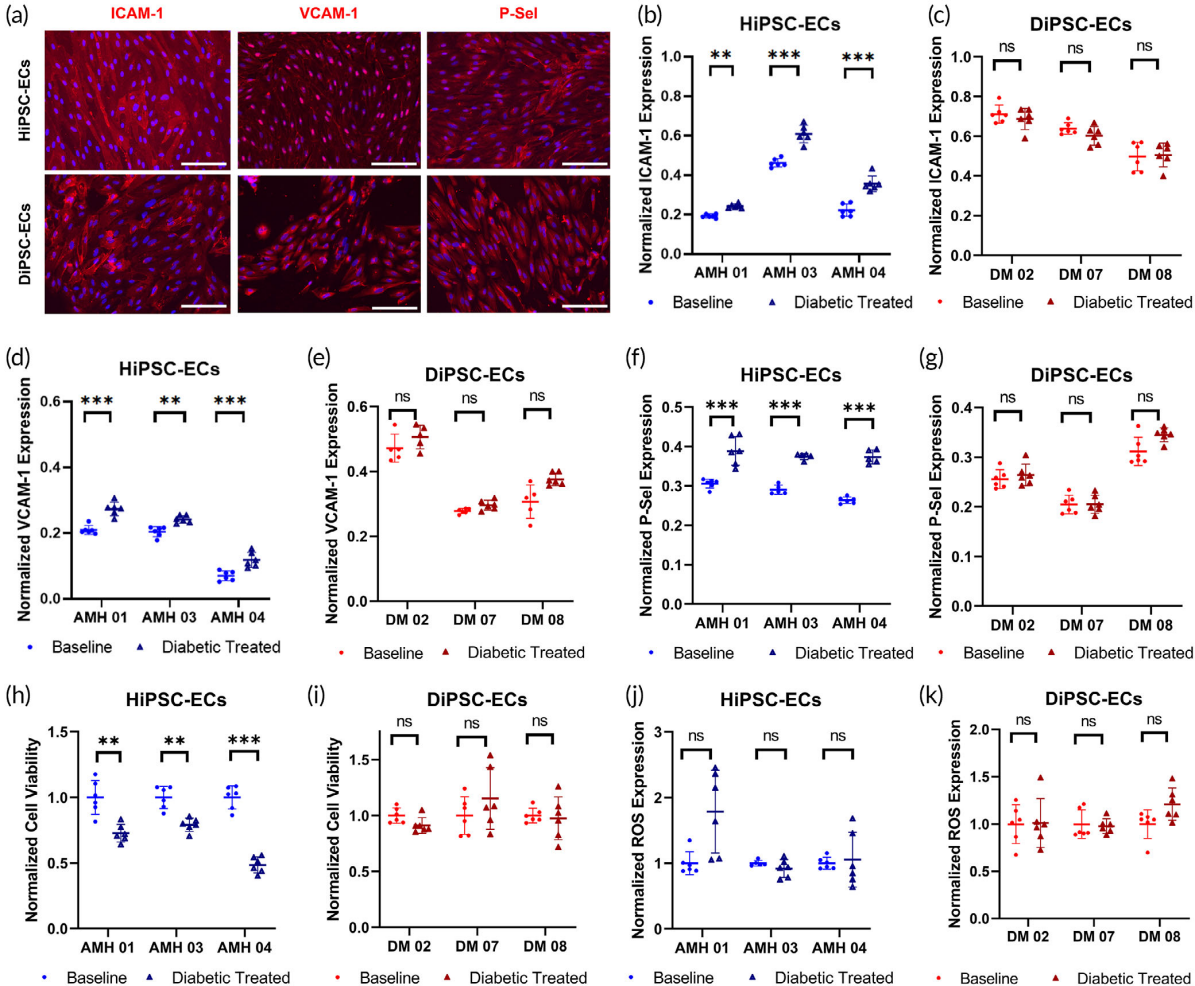
Characterization of HiPSC‐ECs and DiPSC‐ECs after exposure to a diabetogenic extracellular microenvironment. (a) Representative images of HiPSC‐ECs and DiPSC‐ECs staining positive for adhesion molecules (ICAM‐1, VCAM‐1 and P‐Selectin) after 5‐day diabetogenic extracellular treatment; (b) Elevated ICAM‐1 expression observed in HiPSC‐ECs after the diabetic treatment; (c) No significant change in ICAM‐1 expression observed in DiPSC‐ECs after the diabetic treatment; (d) Elevated VCAM‐1 expression observed in HiPSC‐ECs after the diabetic treatment; (e) No significant change in VCAM‐1 expression observed in DiPSC‐ECs after the diabetic treatment; (f) Elevated P‐selectin expression observed in HiPSC‐ECs after the diabetic treatment; (g) No significant change in P‐selectin expression observed in DiPSC‐ECs after the diabetic treatment; (h) Reduction in cell viability observed in HiPSC‐ECs after the diabetic treatment; (i) No significant change in cell viability observed in DiPSC‐ECs after the diabetic treatment; (j) No significant change ROS production observed in HiPSC‐ECs after the diabetic treatment; (k) No significant change ROS production observed in DiPSC‐ECs after the diabetic treatment. Scale bar = 200 μm. *p < 0.05. **p < 0.01. ***p < 0.001. n.s. not significant.

To further evaluate the role of a diabetic‐like microenvironment on the endothelial function of HiPSC‐ECs and DiPSC‐ECs, we treated the cells for 5 days with a diabetogenic medium that contains high glucose (25 mM), urea nitrogen (9 mM), and inflammatory cytokine TNF‐α (20 pg/mL). These conditions were chosen based on clinical serological studies of diabetic patients.^18^, ^19^ After the treatment, HiPSC‐ECs exhibited significant upregulation of adhesion molecules, including ICAM‐1, VCAM‐1, and P‐Selectin (Figure 4a), in comparison to HiPSC‐ECs under normal culture condition (Figure 2a). However, DiPSC‐ECs exhibited limited response after the diabetogenic treatment and maintained the expression of inflammatory markers (Figure 4a), similar to DiPSC‐ECs at the baseline level (Figure 2a). Quantitative measurement of ICAM‐1 expression with in‐cell ELISA demonstrated a significant elevation of ICAM‐1 expression after the diabetogenic treatment for HiPSC‐ECs (Figure 4b). However, no significant difference was detected in DiPSC‐ECs before and after the diabetogenic treatment (Figure 4c). Similar results were observed for expression of VCAM‐1 and P‐selectin (Figure 4d–g). Cell viability after the diabetogenic treatment decreased significantly for HiPSC‐ECs (Figure 4h), but not for DiPSC‐ECs (Figure 4i). This suggests that HiPSC‐ECs are more sensitive toward the environmental change while DiPSC‐ECs have been pre‐conditioned to tolerate the diabetic‐like microenvironment. Lastly, diabetogenic treatment resulted in significant variations in ROS generation for both HiPSC‐ECs (Figure 4j) and DiPSC‐ECs (Figure 4k), without statistically significant difference relative to the baseline ROS. Overall, HiPSC‐ECs were more responsive toward the diabetogenic treatment with significant changes in several of the evaluated parameters compared to the baseline, while DiPSC‐ECs maintained a similar status as the baseline level without significant response to the changes.

### 2.5 Endothelial function of DiPSC‐ECs could be improved by individualized pharmacological treatment

**FIGURE 5:**
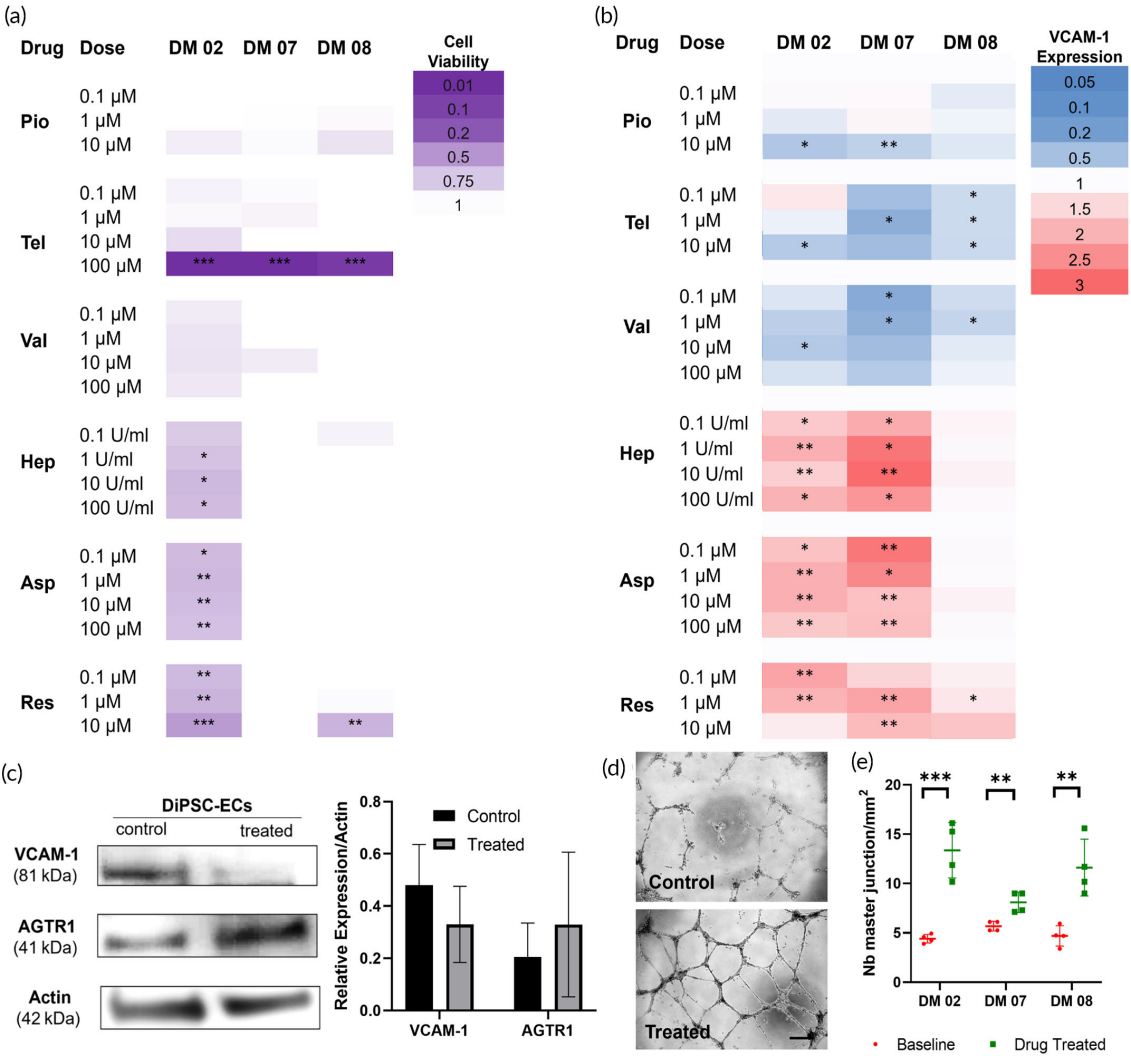
Drug screening with DiPSC‐ECs as in vitro models of diabetic endothelial dysfunction. (a) Heatmap of cytotoxicity at 48 h when DiPSC‐ECs were treated with potentially therapeutic compounds at various concentrations; Purple: high cytotoxicity; White: no cytotoxicity. (b) Heatmap of VCAM‐1 expression relative to cells with no drug treatment when DiPSC‐ECs were treated with potentially therapeutic compounds at various concentrations; Blue: reduced VCAM‐1 expression; White: baseline VCAM‐1 expression; Red: elevated VCAM‐1 expression. (c) Representative western blots and quantitative analysis of DiPSC‐ECs expressing AngII receptor AGTR1 and reduced levels of VCAM‐1 after drug treatment; (d) Re‐evaluation of DiPSC‐EC tubulogenesis after the optimized drug treatment resulted in improved tube formation; (e) Quantification of master junction density in tubulogenesis analysis indicating improved tube formation after drug treatment with DiPSC‐ECs. Pio: pioglitazone; Tel: telmisartan; Val: valsartan; Hep: heparin; Asp: aspirin; Res: resveratrol. *p < 0.05. **p < 0.01. ***p < 0.001.

Next, we explored the use of patient‐derived DiPSC‐ECs as in vitro disease models for diabetic endothelial dysfunction to identify potential pharmacological agents for their functional improvement. Given that DiPSC‐ECs all exhibited significantly elevated adhesion molecule expression relative to HiPSC‐ECs, we performed a small‐scale drug screening for compounds that have been reported to improve EC functions: pioglitazone (a PPAR‐γ agonist),^20^ telmisartan (an angiotensin receptor blocker),^21^ valsartan (an angiotensin receptor blocker),^22^ heparin (an anticoagulant),^23^ aspirin (a nonsteroidal anti‐inflammatory drug and blood thinner),^24^ and resveratrol (an antioxidant polyphenol).^25^ Specifically, we screened for the safety and efficacy in reducing inflammation status via VCAM‐1 expression with those compounds at various doses for each patient's DiPSC‐ECs. Interestingly, the patient‐derived cells exhibited a difference in tolerance toward the drug treatment (Figure 5a and Figure S6). For instance, drugs at all doses, except for telmisartan at 100 μM, were not cytotoxic toward DiPSC‐ECs from DM07, whereas almost all six compounds exhibit dose‐dependent cytotoxicity toward DiPSC‐ECs from DM02. The efficacy in reducing VCAM‐1 expression was mostly consistent among the patients, with a reduction in VCAM‐1 expression when treated with pioglitazone, telmisartan, and valsartan, whereas treatment with heparin, aspirin, or resveratrol resulted in no effect or slight increase in VCAM‐1 expression (Figure 5b and Figure S6). An optimized therapeutic treatment was identified for each patient (DM02: Valsartan at 10 μM; DM07: Telmisartan at 1 μM; DM08: Valsartan at 1 μM) that resulted in a significant reduction in VCAM‐1 expression without causing cytotoxicity. DiPSC‐ECs were treated with individually optimized pharmacological treatment for 48 h and evaluated for their endothelial functional recovery. Western blotting study confirmed reduced VCAM‐1 expression in DiPSC‐ECs after treatment with Angiotensin II receptor blockade, and that Angiotensin II Receptor Type‐1 (AGTR1) is expressed by DiPSC‐ECs with and without pharmacological treatment (Figure 5c). Additionally, DiPSC‐ECs exhibited significantly improved tubulogenesis after each treatment (Figure 5d), including the number of master junctions (Figure 5e) and other parameters evaluated (Figure S7). However, no significant improvement was found in other endothelial functions of DiPSC‐ECs after the pharmacological treatment, such as barrier function or anti‐platelet adhesion.

## 3 DISCUSSION

In this study, we investigated the feasibility of using iPSC technology in combination with diabetogenic treatment to generate in vitro disease models of diabetic endothelial dysfunction. We show that iPSC‐ECs can be successfully generated from the blood of healthy and diabetic patients, where DiPSC‐ECs exhibit impaired endothelial tubulogenic, anti‐thrombotic, and barrier functions with elevated inflammatory status and ROS production, in contrast to the normal endothelial functions of HiPSC‐ECs. This dysfunctional phenotype of DiPSC‐ECs is consistent with the pathophysiology of various diabetic vascular complications, which typically exhibit low angiogenic potential, high risk of thrombosis, and increased inflammation.^26^ It is likely that the functional impairments of DiPSC‐ECs observed in the study reflect the clinical status of the patients at the time of the blood donation. Our results are also consistent with a recent study by Su et al., where ECs differentiated from the iPSCs of two adult patients with type 2 diabetes also displayed disrupted glycine homeostasis, increased senescence, and impaired mitochondrial function and angiogenic potential, in comparison to iPSC‐ECs derived from two healthy individuals.^27^ Taken together, these studies suggest that diabetic endothelial dysfunction can be successfully recapitulated in vitro with iPSC technology.

Previous in vitro models of diabetic endothelial dysfunction mostly utilized hyperglycemia or other diabetic‐like conditions to stimulate healthy ECs to induce a diabetic‐like phenotype.^28^ Intriguingly, our results indicate that a diabetic‐like microenvironment is not necessary to induce diabetic‐like phenotypes for DiPSC‐ECs. This is also consistent with a previous study of diabetic cardiomyopathy by Drawnel et al., where cardiomyocytes derived from diabetic patient‐specific iPSCs exhibited a cardiomyopathic phenotype in the absence of a diabetic stimulus.^29^ These results suggest that cells derived from diabetic patients with iPSC technology are genetically and/or epigenetically predisposed to dysfunction related to diabetes. Genetically, meta‐analyses of genome‐wide association studies (GWAS) have identified more than 240 loci that are associated with type 2 diabetes (T2D).^30^ Those genetic variations, if present in the diabetic patients in our study, will be retained during the reprogramming and differentiation processes from patient blood into DiPSC‐ECs. Additionally, glycemic memory‐associated epigenetic changes such as DNA methylation have been postulated to play a role in the pathogenesis and progression of diabetic complications.^31^ Previous studies have indicated that iPSCs reprogrammed by Yamanaka factors harbor residual DNA methylation signatures characteristic of their somatic tissue of origin.^32^, ^33^ Therefore, it is likely that iPSCs generated from diabetic patients in our study also retain some of their epigenetic memory as a consequence of epigenetically modified gene‐expression patterns. Future studies will investigate the roles of genetic and epigenetic factors contributing to the diabetic endothelial dysfunction of DiPSC‐ECs. Our analysis with bulk RNA sequencing reveals several transcriptional differences between healthy and disease patients, both in iPSCs and iPSC‐ECs. Since even iPSCs from a single individual can result in highly heterogeneous differentiated populations,^34^ future single‐cell studies can unravel within‐sample and across‐sample differences between individual stem or differentiated cells within a population.

In this study, we also recreated the complex microenvironment of diabetes in vitro by exposing iPSC‐ECs to hyperglycemia, elevated inflammation, and hyper‐urea levels, which are characteristic features of the diabetic condition. Although previous models have primarily used hyperglycemia as the only condition replicated, our approach more closely reflects the complex nature of the diabetic microenvironment. For example, approximately one in three adults with diabetes have been diagnosed with chronic kidney disease,^35^ resulting in elevated levels of blood urea above 25 mg/dL.^18^ Chronic inflammation and elevated levels of TNFα have been shown to be associated with diabetes.^36^ It was found that diabetic patients have significantly higher serum levels of the inflammatory TNFα.^36^ Therefore, to recapitulate the complexity of diabetic microenvironment, we have incorporated hyperglycemia, hyper‐urea, and elevated inflammation in our system. Interestingly, we found that an acute 5‐day exposure to diabetic conditions was sufficient to induce a dysfunctional phenotype in healthy iPSC‐ECs, while the same treatment did not affect the already dysfunctional diabetic iPSC‐ECs, suggesting potential epigenetic memory. Our approach of incorporating multiple relevant disease conditions may provide a more accurate model for understanding the complex pathophysiology of diabetic endothelial dysfunction and aid in the identification of novel therapeutic targets.

We also show that DiPSC‐ECs could be used as an in vitro screening platform for diabetic endothelial dysfunction. Suppression of the pro‐inflammatory phenotype was used as a screening endpoint to identify molecules that improve the endothelial function of DiPSC‐ECs. Currently, no specific drug treatment exists to prevent or rescue diabetes‐induced cardiovascular complications clinically. Clinical treatment options typically aim to establish glycemic control and delay the progression of the disease.^37^ As endothelial dysfunction is a key mediator for diabetic vascular complications, therapeutics that aim to restore endothelial function in diabetes could potentially improve the clinical outcomes for those patients. To that end, a number of promising therapeutic candidates have been identified in preclinical studies using various animal models of diabetes. However, many of the promising therapeutic candidates with success in preclinical evaluations have failed to show efficacy in human randomized control trials. One possible reason for this inability to translate findings to patients could be due to the inability of the preclinical animal models to recapitulate the clinical condition. In vitro humanized disease models, known as “disease‐in‐a‐dish,” have a strong potential to fill the gap between preclinical studies and clinical trials. The iPSC technology enables creating disease‐specific cellular models, investigating underlying mechanisms surrounding different diseases, and optimizing therapy and treatment options for patients.^38^

As demonstrated by our study, DiPSC‐ECs can serve as in vitro human models of diabetic endothelial dysfunction. Although our current study only uses a limited amount of patient samples (*n* = 3), we have shown the functional differences between healthy control subjects and diabetic patients are consistent across all the patient samples analyzed. As iPSCs have the ability to continuously proliferate, this will also provide a viable cell source for large‐scale, high‐throughput screening in future drug discovery. Furthermore, we identified individualized treatment with the ability to improve the phenotype and function of DiPSC‐ECs from each patient at least partially. Diabetes is a complex, polygenic condition with known contributions of lifestyle and genetics and significant variations among individual patients. Therefore, the traditional “one‐size‐fits‐all” approach may not be feasible in addressing the challenge. Precision medicine approaches such as pharmacogenomics that are common now in cancer treatment are likely needed for safe and effective treatment of diabetic vascular complications in the future. We acknowledge that our study is based on a small cohort of diabetic patients, with an unbalanced gender distribution. Further studies with a larger cohort of patients are needed to validate our findings and to generalize the results to a broader diabetic human population.

Interestingly, the pharmacological agents identified in our study to significantly improve endothelial function in DiPSCs‐ECs, namely valsartan and telmisartan, are both classified as Angiotensin II (AngII) receptor blockers (ARBs). AngII (Asp‐Arg‐Val‐Tyr‐Ile‐His‐Pro‐Phe) is a peptide hormone that is part of the renin–angiotensin–aldosterone system (RAAS) to regulate blood pressure. ECs express AngII receptor type I (AGTR1), which upon activation, initiates an inflammatory cascade of NAD(P)H oxidase activation and mitochondrial ROS generation, leading to increased expression of adhesion molecules (e.g., VCAM‐1, ICAM‐1).^39^ Therefore, pharmacological blockade of AT1 with valsartan or telmisartan in DiPSC‐ECs is expected to effectively reduce VCAM‐1 expression, as demonstrated in our study. ARBs have been used in diabetic patients to treat hypertension and to reduce the rate of diabetic nephropathy progression.^40^ However, ARBs are not routinely used to treat other vascular complications of diabetes, such as ischemic heart disease, stroke, retinopathy, and diabetic foot ulcers. It is likely that the administration of ARBs could be beneficial to diabetic patients with various vascular complications, given our results in the reduction of inflammatory markers and the improvement of tubulogenesis in DiPSC‐ECs. For example, a recent clinical report indicates that ARB use is independently and positively associated with wound healing in patients with diabetic foot ulcers.^41^ Previous preclinical studies also demonstrated the topical application of valsartan accelerated wound healing in diabetic mice and porcine models.^42^ Taken together, these evidence suggest a potential benefit of expanding the use of ARBs to improve vascular functions in diabetes.

### 3.1 Statement of novelty

While the reprogramming and differentiation of iPSCs from diabetic patients have been previously reported in the literature,^27^ our study contributes to the field by providing new insights and data that expand upon previous findings. First, we have performed transcriptome analysis of iPSCs and iPSC‐ECs to compare the global gene expression between diabetic and healthy cells, which has not been reported in previous studies to the best of our knowledge. Our analysis revealed several differentially expressed genes that are involved in endothelial dysfunction and diabetes‐related pathways, which provide insights into the mechanisms underlying diabetic vascular complications. Moreover, we have also used diabetic iPSC‐ECs as a disease model for drug screening and identified individualized pharmacological treatments for each patient cell line. This approach provides a more personalized and precise approach to drug discovery for diabetic vasculopathy, which has not been explored in previous studies. This information could be valuable for developing new therapeutic strategies for diabetic vasculopathy.

## 4 MATERIALS AND METHODS

### 4.1 Human subjects

This study was approved by the Northwestern University Institutional Review Board (Protocol ID STU00209880). All consents and blood collection procedures were performed at the Northwestern Memorial Hospital (Chicago, IL). Ten milliliter of anticoagulant whole blood was obtained from each patient using the following inclusion criteria: age 50–80, fasting plasma glucose (FPG) ≥ 126 mg/dL (7.0 mmol/L), A1C ≥ 6.5%, or a blood glucose level of 200 mg/dL or higher at 2 h per oral glucose tolerance test (OGTT). The patients were retrospectively selected from a larger study on peripheral artery disease (PAD), and all patients have one or more cardiovascular complications including PAD. ABI < 0.9 and Rutherford 3, 4, 5, or 6 scores were used as criteria for inclusion of patients with PAD in the previous study. For age‐matched healthy control (AMH) population, there should be at the same age range with no medical history of diabetes or other cardiovascular complications. The human subject information is listed in Table 1. Approximately 10 mL of blood was obtained by venipuncture of the forearm and collected into sterile acid citrate dextrose (ACD) anticoagulant tubes. Peripheral blood mononuclear cells (PBMCs) were isolated from anticoagulant whole blood on the same day of blood draw using ACCUSPIN™ System‐Histopaque®‐1077 (Sigma‐Aldrich, St. Louis, MO), following manufacturer's protocol.

### 4.2 Reprogramming of PBMCs into iPSCs

All reagents used in this study were obtained from Thermo Fisher Scientific (Waltham, MA) unless otherwise specified. Approximately 2 million PBMCs per subject were used for iPSC reprogramming, using CytoTune™‐iPS 2.0 Sendai Reprogramming Kit, following the user manual “Reprogramming Peripheral Blood Mononuclear Cells (PBMCs) with the CytoTune®‐iPS 2.0 Reprogramming Kit under feeder‐free conditions (Revision 1.0)”. The transduced cells were plated onto recombinant human vitronectin (rhVTV)‐coated surfaces and cultured with the Essential 8 Flex medium system. Colonies were picked at approximately 3 weeks after viral transduction, passaged using 0.5 mM EDTA, and expanded on rhVTN‐coated surfaces with Essential 8 Flex culture medium. G‐banded karyotype analysis was routinely performed for each iPSC line by Cell Line Genetics (Madison, WI).

### 4.3 Embryoid body assay

Patient iPSC colonies were transferred into ultra‐low attachment multi‐well plates in Iscove's Modified Dulbecco's Medium supplemented with 15% fetal bovine serum to form embryoid bodies. After 8 days in suspension culture, the formed embryoid bodies were transferred into 0.1% gelatin‐coated 24‐well plates and cultured for an additional 8 days as adherent culture. The adherent embryoid bodies were fixed with 4% formaldehyde on day 16 and probed for markers of the three germ layers.

### 4.4 Directed differentiation into ECs

Differentiation of iPSC into ECs was performed following a protocol published by Patsch et al.^16^ Briefly, iPSCs were seeded onto rhVTN‐coated surfaces and treated with Essential 8 Flex medium containing ROCK inhibitor Y‐27632 (10 μM) overnight, followed by a 3‐day lateral mesoderm induction using an N2B27 medium containing CHIR‐99021 (8 μM) and rhBMP4 (25 ng/mL), and finished with a 2‐day endothelial induction StemPro‐34 SFM medium supplemented with rhVEGF (200 ng/mL) and forskolin (2 μM). On day 6, all cells were enzymatically detached with trypsin/EDTA and sorted for VE‐Cadherin positive cells with magnetic activated cell sorting (MACS) (Miltenyi Biotec, Auburn, CA). VE‐Cadherin^+^ cells were plated onto human fibronectin‐coated surfaces and supplemented with StemPro‐34 SFM medium with rhVEGF (50 ng/mL). After culture for 5 days, iPSC‐ECs were sub‐culture at 50,000 cells/cm^2^ on fibronectin‐coated surfaces with VascuLife VEGF Endothelial Medium Complete Kit (Lifeline Cell Technologies) for further analysis.

### 4.5 Diabetogenic treatment

To mimic the diabetic microenvironment, patient‐derived iPSC‐ECs were cultured with 25 mM of glucose, 9 mM of urea, and 20 pg/mL of TNF‐ α. Diabetogenic treatment lasted for 5 days with media changes every other day.

### 4.6 Immunofluorescence

Cells were washed with PBS, fixed with 4% formaldehyde, permeabilized with 0.1% Triton‐X and blocked with 0.5% bovine serum albumin (BSA). The cells were then incubated with primary antibodies at 4°C overnight, followed by secondary antibodies at room temperature for 1 h. Cell nuclei were counter‐stained with Hoechst 33258, and all images were acquired via fluorescence microscopy (Nikon TE2000U, Melville, NY, USA). All antibody manufacturer information and dilutions were listed in Table S1.

### 4.7 Flow cytometry

Cells were dissociated into single‐cell suspension with TrypLE, followed by incubation with 1% knockout serum replacement on ice. All primary antibodies labeled with fluorophore were incubated with cells for 20 min on ice, before washing three times with cold PBS. Flow cytometry was performed with BD LSR II (BD Biosciences, Franklin Lakes, NJ), and the data were analyzed with FlowJo_V10 (FlowJo, Ashland, OR) for three independent experiments per cell line (*n* = 3). All antibody manufacturer information and dilutions are listed in Table S1.

### 4.8 Bulk sequencing and analysis

Bulk paired‐end (61:8:8:61) RNA sequencing was done using MiRNeasy Micro Kit (Qiagen 217,084) for extraction, NEBNext® Poly(A) mRNA Magnetic Isolation Module (E7490L), NEBNext® Ultra™ II RNA Library Prep Kit for Illumina® (E7770L), and NEBNext® Multiplex Oligos for Illumina® (Dual Index Primers Set 1) (E7600S), as described previously.^43^, ^44^ Prior to library preparation, the samples were randomized to avoid experimental and human biases. We started our experiments with a total of 24 samples (iPSCs and differentiated iPSCs‐ECs) from healthy and disease patients. During library preparation, two DiPSC and 2 HiPSC‐EC patient samples were excluded from sequencing due to insufficient RNA isolation. RNA‐seq reads were pseudo‐aligned to the human transcriptome (GRCH38 cDNA) utilizing Kallisto v0.48.0 to generate estimated gene count matrices.^45^ Count matrices were normalized and differential gene expression analyses were done with DESeq2 v1.38.3 using R v4.2.3 for each subgrouping comparison.^46^ The count matrix was further filtered for low expression counts such that any gene less than a total of 10 counts across samples cutoff were removed from downstream analysis. To compare between samples, differential gene expression data was extracted. The top 50 most up‐ and downregulated genes (total 100, unless the number of differentially expressed genes were <100) were filtered utilizing an ordered Log2FC gene list and then used to generate corresponding comparisons within heat maps. GO analysis was performed using *clusterProfiler* in R. Scripts for all extraction and downstream differential expression analysis are provided at the Goyal laboratory GitHub: https://github.com/GoyalLab/Bulk-RNA-Jiang-Analysis.git. Raw data are uploaded to the NCBI GEO repository with accession number GSE236430 and are available to the public.

### 4.9 Western blotting

Cells were grown on 6‐well plates and lysed in RIPA buffer supplemented with protease inhibitors. Total protein concentration was determined using a BSA assay kit. Equal amounts of protein per sample (5 μg) were separated on a 4%–12% Bis‐Tris gel by electrophoresis and transferred onto a nitrocellulose membrane. The membrane was then blocked with BSA and incubated with the primary antibodies overnight at 4°C. After washing with PBS‐T, the membrane was incubated with secondary antibodies conjugated to horseradish peroxidase (HRP) for an hour at room temperature. Protein bands were visualized after reacting with a chemiluminescent substrate and imaged with Azure c300 Gel Imager (Azure Biosystems, Dublin, CA). Quantitative analysis was performed with ImageJ. All antibody information and dilutions are listed in Table S1.

### 4.10 Cell viability assessment

A resazurin‐based assay was used to assess cell viability after diabetic microenvironment treatment. On day 5 of the diabetic microenvironment treatment, iPSC‐ECs were incubated with resazurin (0.025 mg/mL, Sigma, R7017) for 1.5 h before fluorescence was read at 560/590 nm on the Cytation 3 microplate reader (BioTek). Readings for quantitative assays were adjusted to the cell viability per experiment.

### 4.11 In‐cell enzyme‐linked immunosorbent assay (ELISA)

For quantitative analysis of cellular expression of proteins in iPSC‐ECs, the cells were seeded onto a 96‐well plate with fibronectin coating at 5000 cells/well. After treatments, the cells were fixed with 4% formaldehyde, permeabilized with 0.1% Triton‐X and blocked with 5% FBS. Primary antibodies were incubated with each well at 4°C while the secondary antibody with HRP conjugate was incubated at room temperature for 1 h. 3,3′,5,5′‐tetramethylbenzidine (TMB, 100 μL/well) was added to each well for 20 min, before stopped with 0.16 M sulfuric acid (50 μL/well). Absorbance at 450 nm was read with Cytation 5. All readings were normalized to cell numbers based on resazurin assay.

### 4.12 Platelet adhesion

Human platelets were isolated from anticoagulant whole blood samples of healthy subjects, and diluted to 10^8^ platelets mL^−1^ in platelet suspension buffer as previously described.^47^ The platelet suspension was incubated at 37°C for 30 min with monolayers of patient‐specific iPSC‐ECs. The adherent platelets were probed with platelet‐specific antibody CD41, while the patient ECs were counter‐stained with CD144 and Hoechst 33258. The images were acquired with Cytation 5 Cell Imaging Multi‐Mode Reader (BioTek Instruments, Winooski, VT) and analyzed with Gen5 Data Analysis Software (BioTek). A total of four images were acquired for each well, and a total of 4 wells were analyzed for each patient (*n* = 4).

### 4.13 Tubulogenesis

Matrigel matrix (125 μL/well) was added to a 48‐well plate and incubated at 37°C to form gels. Patient‐specific iPSC‐ECs at passage 2 were dissociated into single‐cell suspension and seeded onto the matrix at 40,000/well in EC medium, with four wells per patient (*n* = 4). Eighteen hours after cell seeding, images were taken with phase contrast microscopy (Nikon TE2000U, Melville, NY) with a 4× objective. All images were analyzed with ImageJ with the Angiogenesis Analyzer plugin.

### 4.14 Permeability assay

A transwell permeability assay was used to gain a quantitative understanding of the iPSC‐EC monolayer's barrier function. iPSC‐ECs were seeded directly onto transwell cell culture inserts (0.4 um pore size) at a seeding density of 50,000 k/cm^2^. Horseradish peroxidase (HRP, 0.125 μM, ProspecBio) was added to the top chamber of transwell insert and incubated with the endothelial monolayer for 45 min, after which samples were obtained from the lower chamber to react with TMB solution. Absorbance was read at 450 nm after adding stop solution.

### 4.15 Reactive oxygen species generation assay

The intracellular generation of reactive oxygen species (ROS) was measured with the oxidative stress indicator CM‐H_2_DCFDA (chloromethyl derivative of 2′,7′‐di‐chlorofluorescein diacetate). Manufacturer recommended protocol was closely followed. Briefly, CM‐H_2_DCFDA was diluted to a final concentration of 10 μM in pre‐warmed PBS supplemented with calcium and magnesium for 60 min, before obtaining fluorescent measurements at 485/528 nm.

### 4.16 Nitric oxide generation assay

The intracellular production of nitric oxide (NO) was measured with the nitric oxide indicator, DAF‐FM diacetate (4‐amino‐5‐methylamino‐2′,7′‐difluorescein diacetate). Manufacturer recommended protocol was closely followed. Briefly, DAF‐2‐DA (1 μM in PBS) was incubated with 50,000 iPSC‐ECs/well in a 24‐well plate for 30 min, and the fluorescence images were taken at 485/528 nm. To measure nitric oxide released to the extracellular environment, the cell culture medium (24 h after cell seeding) was collected, and the nitrate was converted to nitrite with nitrate reductase/NADPH. Total nitrite concentration was detected with a Measure‐IT High‐Sensitivity Nitrite Assay Kit and normalized to cell number using a Quant‐iT PicoGreen dsDNA Assay Kit, following manufacturer's instructions.

### 4.17 Drug screening

DiPSC‐ECs were seeded onto fibronectin coated 96‐well plate at 5000 cells/well. Pioglitazone, resveratrol, valsartan, telmisartan and aspirin (Sigma Aldrich, St. Lois, MO) at 0.1, 1, 10 and 100 μM, and heparin sodium (Celsus, Cincinnati, OH) at 0.1, 1, 10, and 100 U mL^−1^ were incubated with DiPSC‐ECs for 48 h. Cell viability was assessed via resazurin assay and VCAM‐1 expression was assessed via cell‐based ELISA. All readings from resazurin assay were normalized to no drug treatment as 1. All readings from cell‐based ELISA were then normalized to cell viability based on the resazurin assay from the same plate. Optimized drug and dosage were identified for each patient cell line based on cytotoxicity and VCAM‐1 expression and were used to treat each cell lines for 48 h. Tubulogenesis was re‐evaluated with and without optimized drug treatment as previously described.

### 4.18 Statistical analysis

Three subjects (*n* = 3, biological replicates) were included in each group. For each experiment, *n* ≥ 3 experimental replicates were carried out per biological replicate. All data were plotted and analyzed with GraphPad Prism 9.2.0 (GraphPad Software, San Diego, CA, USA). For all statistical analysis, a normality test was performed to confirm normal distribution prior to statistical comparison. Nested 2‐tail *t*‐tests were used to compare between the diabetic patient group and the healthy control group, and *p* < 0.05 was considered statistically significant. Paired t‐tests were used to compare the diabetic microenvironment treatment to untreated cells within the same cell line, and *p* < 0.05 was considered statistically significant. For the drug screening study, a heatmap was plotted after normalization to the baseline. One‐way ANOVA with Tukey post hoc test were used for each cell line individually, and *p* < 0.05 was considered statistically significant.

## 5 CONCLUSIONS

In conclusion, we have evaluated the function of both healthy and diabetic patient‐derived iPSC‐ECs under normal and diabetic conditions while considering inflammation, oxidative stress, permeability, and thrombosis. Overall, we observed a pro‐inflammatory and pro‐thrombotic phenotype in cells derived from diabetic patients, with implications in the progression of atherogenesis and thrombosis. Exposure to a diabetic‐like microenvironment induced a phenotypic surrogate of diabetic endothelial dysfunction in cells obtained from healthy subjects but did not have an effect on the cells derived from diabetic patients. Our in vitro disease models, which consider the complexity of genetic and environmental factors in diabetes, provide a screening platform for identifying candidate molecules that can rescue the disease phenotype. Future work will focus on expanding the drug screening to a high‐throughput platform using our in vitro models to identify therapeutics that could restore or prevent the dysfunctional phenotype. Moreover, our disease model will be leveraged to provide mechanistic insights into the underlying pathophysiology of diabetic endothelial dysfunction. This platform is expected to bridge the gap between preclinical animal studies and human clinical trials, allowing for reduced patient response stratification and guiding the future clinical management of diabetic vascular complications.

## AUTHOR CONTRIBUTIONS

**Rayyan Gorashi:** Conceptualization (equal); data curation (lead); formal analysis (lead); investigation (lead); methodology (lead); writing – original draft (lead). **Nancy Rivera‐Bolanos:** Data curation (equal); formal analysis (equal); investigation (equal); methodology (equal); validation (equal); writing – review and editing (equal). **Caitlyn Dang:** Data curation (equal); investigation (equal); methodology (equal). **Cedric Chai:** Formal analysis (equal); investigation (supporting); methodology (supporting); software (lead); writing – review and editing (supporting). **Beatrix Kovacs:** Data curation (equal); investigation (equal); methodology (equal); supervision (equal); writing – review and editing (equal). **Sara Alharbi:** Data curation (equal); methodology (equal); validation (equal); writing – review and editing (equal). **Syeda Subia Ahmed:** Data curation (supporting); methodology (supporting). **Yogesh Goyal:** Conceptualization (equal); investigation (equal); writing – original draft (equal); writing – review and editing (equal). **Guillermo Ameer:** Conceptualization (equal); investigation (equal); writing – review and editing (equal). **Bin Jiang:** Conceptualization (equal); data curation (equal); formal analysis (equal); funding acquisition (equal); investigation (equal); methodology (equal); project administration (equal); resources (equal); software (equal); supervision (equal); validation (equal); visualization (lead); writing – original draft (equal); writing – review and editing (lead).

## CONFLICT OF INTEREST STATEMENT

The authors have no conflict of interest related to this work to disclose.

### PEER REVIEW

The peer review history for this article is available at https://www.webofscience.com/api/gateway/wos/peer-review/10.1002/btm2.10592.

## Supporting information


**Figure S1.** G‐banded karyotypical analysis for all iPSCs. The karyotyping was conducted for HiPSCs and DiPSCs at passages between 7 and 15 by cell line genetics (Madison, WI).
**Figure S2.** Principal component analysis (PCA) plot of iPSCs and iPSC‐ECs across both healthy individuals and diabetic patient samples.
**Figure S3.** Whole genome volcano plot of iPSCs and iPSC‐ECs (Log2fold threshold = 2, adjusted *p* value threshold = 0.01).
**Figure S4.** Whole genome volcano plot of HiPSC‐ECs and DiPSC‐ECs (Log2fold threshold = 2, adjusted *p* value threshold = 0.01).
**Figure S5.** Whole genome volcano plot of HiPSCs and DiPSCs (Logfold2 threshold = 2, adjusted *p* value threshold = 0.01).
**Figure S6.** Cytotoxicity via resazurin assay and inflammatory status assessment via VCAM‐1 ELISA of each diabetic patient's DiPSC‐ECs when treated with screened drugs at various doses for 48 h. # not available; * *p* < 0.05; ** *p* < 0.01; *** *p* < 0.001
**Figure S7.** Heatmap of the parameters measured in the tubulogenesis assay after treated with the optimized drug for each line of DiPSC‐ECs, relative to the cells without the drug treatment. * *p* < 0.05; ** *p* < 0.01; *** *p* < 0.001

**Table S1.** DNA fingerprinting STR profiles of PBMCs, iPSCs and iPSC‐ECs from each human subject by Cell Line Genetics (Madison, WI).

**Table S2.** RNA‐seq differential gene expression analysis of HiPSCs and HiPSC‐ECs. DESeq2 Gene count table showing differentially expressed genes between HiPSC and HiPSC‐EC samples.

**Table S3.** RNA‐seq differential gene expression analysis of HiPSC‐ECs and DiPSC‐ECs. DESeq2 Gene count table showing differentially expressed genes between HiPSC‐EC and DiPSC‐EC samples.

**Table S4.** RNA‐seq differential gene expression analysis of HiPSCs and DiPSCs. DESeq2 Gene count table showing differentially expressed genes between HiPSC and DiPSC‐ samples.

**Table S5.** List of antibodies used in this study for immunofluorescence imaging (i), flow cytometry (f), western blotting (w), and cell‐based ELISA (e).

## Data Availability

The RNA‐seq data that support the findings of this study are openly available from NCBI Gene Expression Omnibus (GEO) with accession number GSE236430 at https://www.ncbi.nlm.nih.gov/geo/query/acc.cgi?acc=GSE236430. Other data that support the findings of this study are available from the corresponding author upon reasonable request.
