# Supplementary material for: Modeling diabetic endothelial dysfunction with patient‐specific induced pluripotent stem cells
Source: Bioeng Transl Med. 2023 Aug 30;8(6):e10592. doi: 10.1002/btm2.10592 (PMC10658533; doi:10.1002/btm2.10592)
Supplement: Supplementary file 1 — Figure S1. G‐banded karyotypical analysis for all iPSCs. The karyotyping was conducted for HiPSCs and DiPSCs at passages between 7 and 15 by cell line genetics (Madison, WI). Figure S2. Principal component analysis (PCA) plot of iPSCs and iPSC‐ECs across both healthy individuals and diabetic patient samples. Figure S3. Whole genome volcano plot of iPSCs and iPSC‐ECs (Log2fold threshold = 2, adjusted p value threshold = 0.01). Figure S4. Whole genome volcano plot of HiPSC‐ECs and DiPSC‐ECs (Log2fold threshold = 2, adjusted p value threshold = 0.01). Figure S5. Whole genome volcano plot of HiPSCs and DiPSCs (Logfold2 threshold = 2, adjusted p value threshold = 0.01). Figure S6. Cytotoxicity via resazurin assay and inflammatory status assessment via VCAM‐1 ELISA of each diabetic patient's DiPSC‐ECs when treated with screened drugs at various doses for 48 h. # not available; * p < 0.05; ** p < 0.01; *** p < 0.001 Figure S7. Heatmap of the parameters measured in the tubulogenesis assay after treated with the optimized drug for each line of DiPSC‐ECs, relative to the cells without the drug treatment. * p < 0.05; ** p < 0.01; *** p < 0.001 [file BTM2-8-e10592-s003.docx]

**
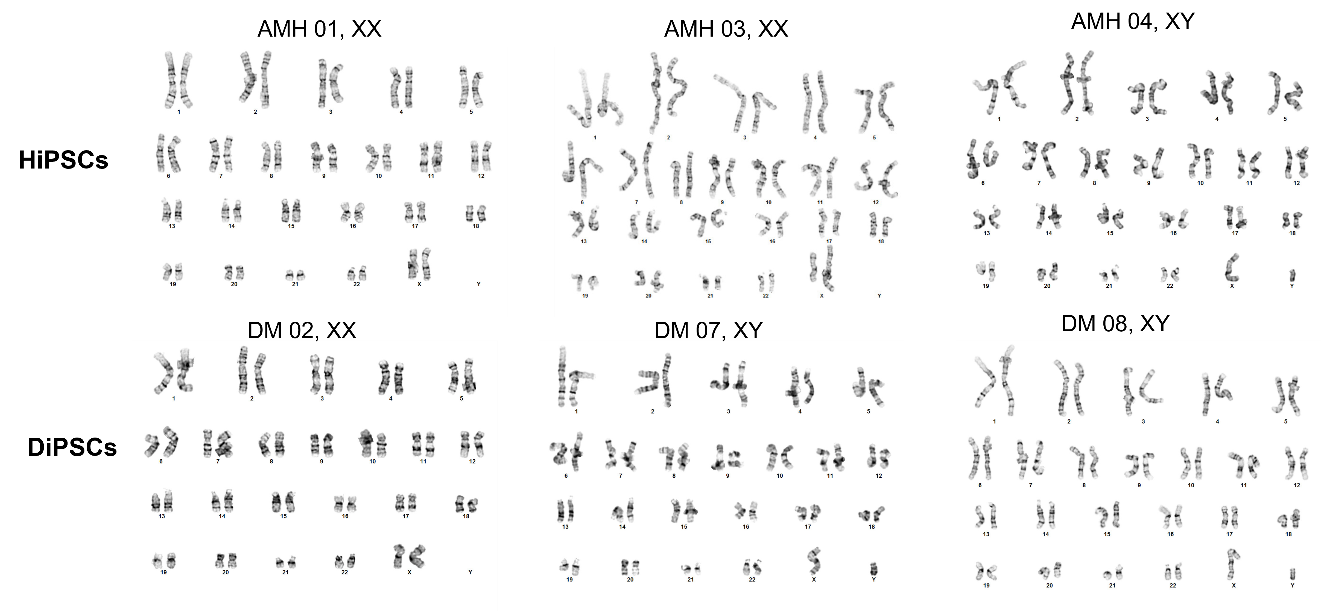
**

**Supplemental Figure S1.** G-banded karyotypical analysis for all iPSCs. The karyotyping was conducted for HiPSCs and DiPSCs at passages between 7 and 15 by Cell Line Genetics (Madison, WI).


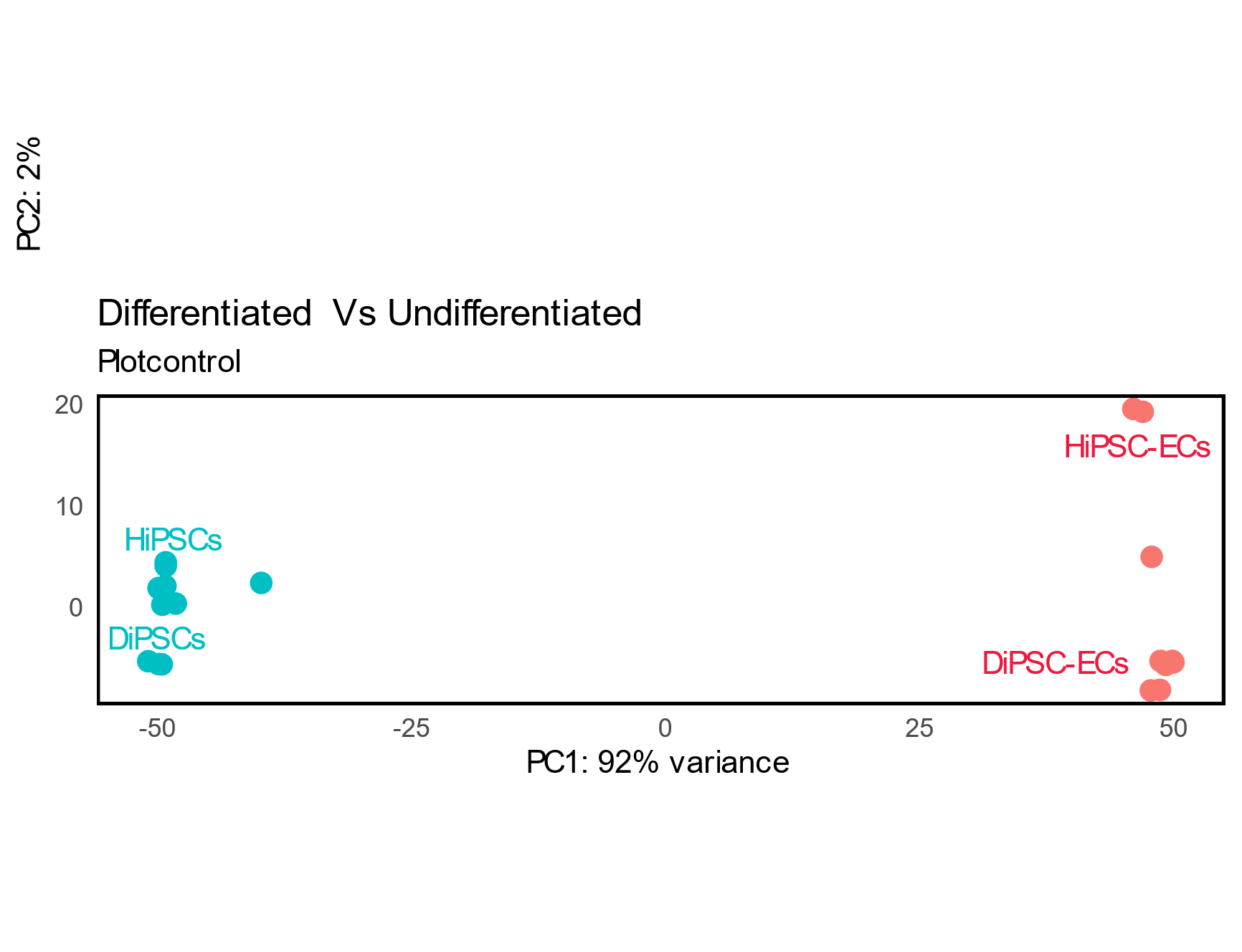


**Supplemental Figure S2.** Principal component analysis (PCA) plot of iPSCs and iPSC-ECs across both healthy individuals and diabetic patient samples.


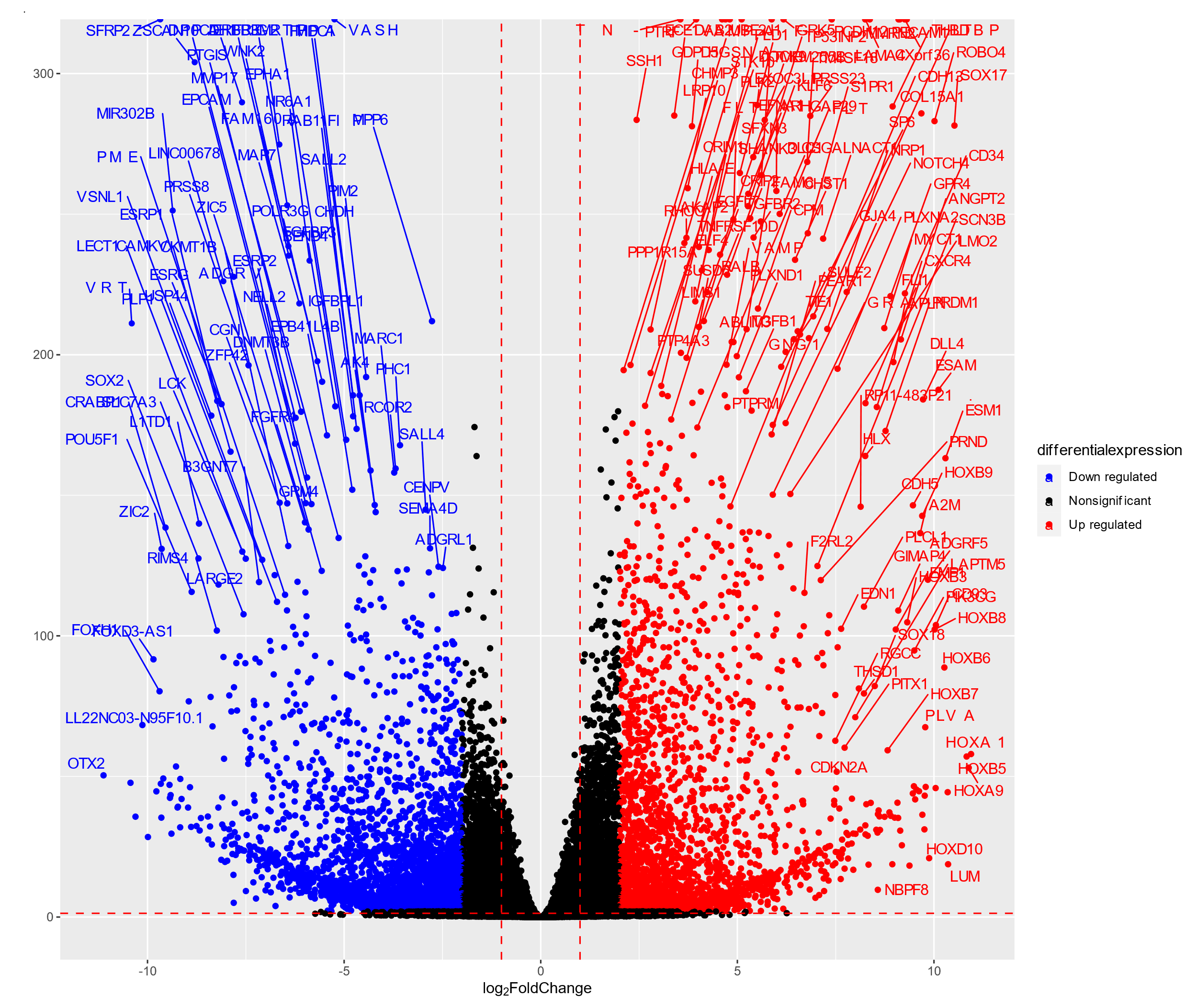


**Supplemental Figure S3.** Whole genome volcano plot of iPSCs and iPSC-ECs (Log2fold threshold = 2, adjusted P-value threshold = 0.01).


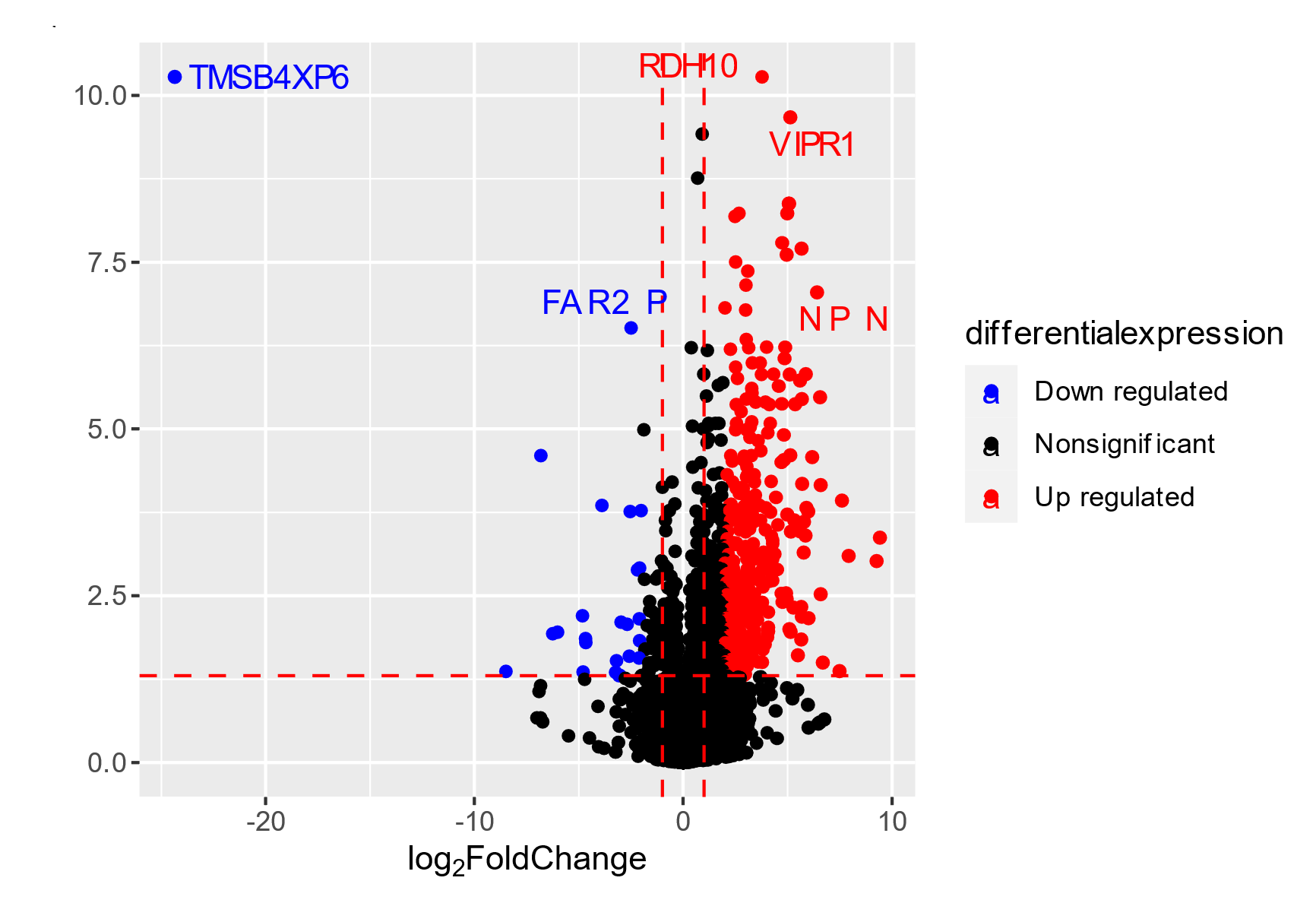


**Supplemental Figure S4.**  Whole genome volcano plot of HiPSC-ECs and DiPSC-ECs (Log2fold threshold = 2, adjusted P-value threshold = 0.01).


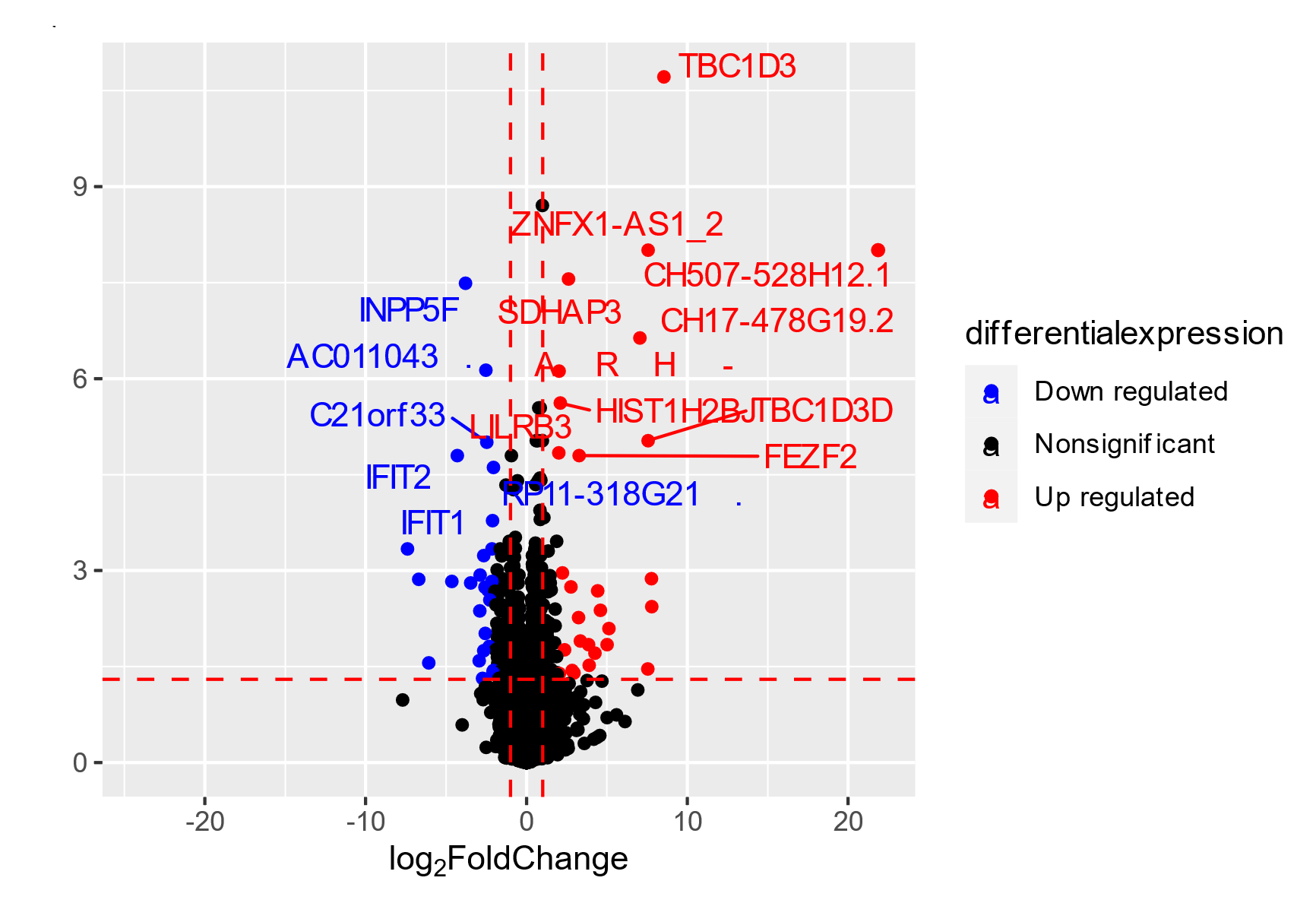


**Supplemental Figure S5.**  Whole genome volcano plot of HiPSCs and DiPSCs (Logfold2 threshold = 2, adjusted P-value threshold = 0.01).


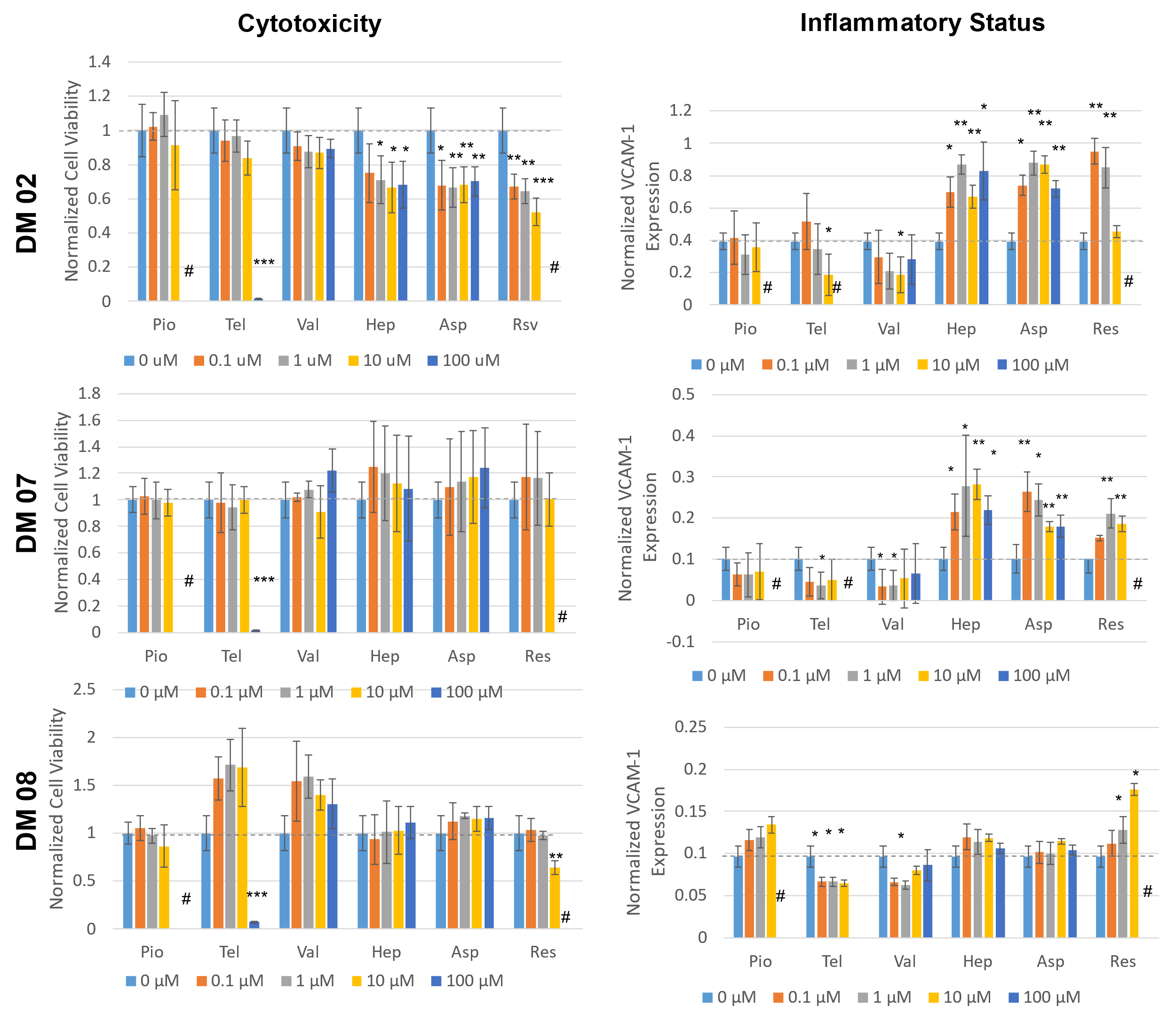


**Supplemental Figure S6**. Cytotoxicity via resazurin assay and inflammatory status assessment via VCAM-1 ELISA of each diabetic patient’s DiPSC-ECs when treated with screened drugs at various doses for 48 hours.  **#** not available; ***** p<0.05; ** p<0.01; *** p<0.001**
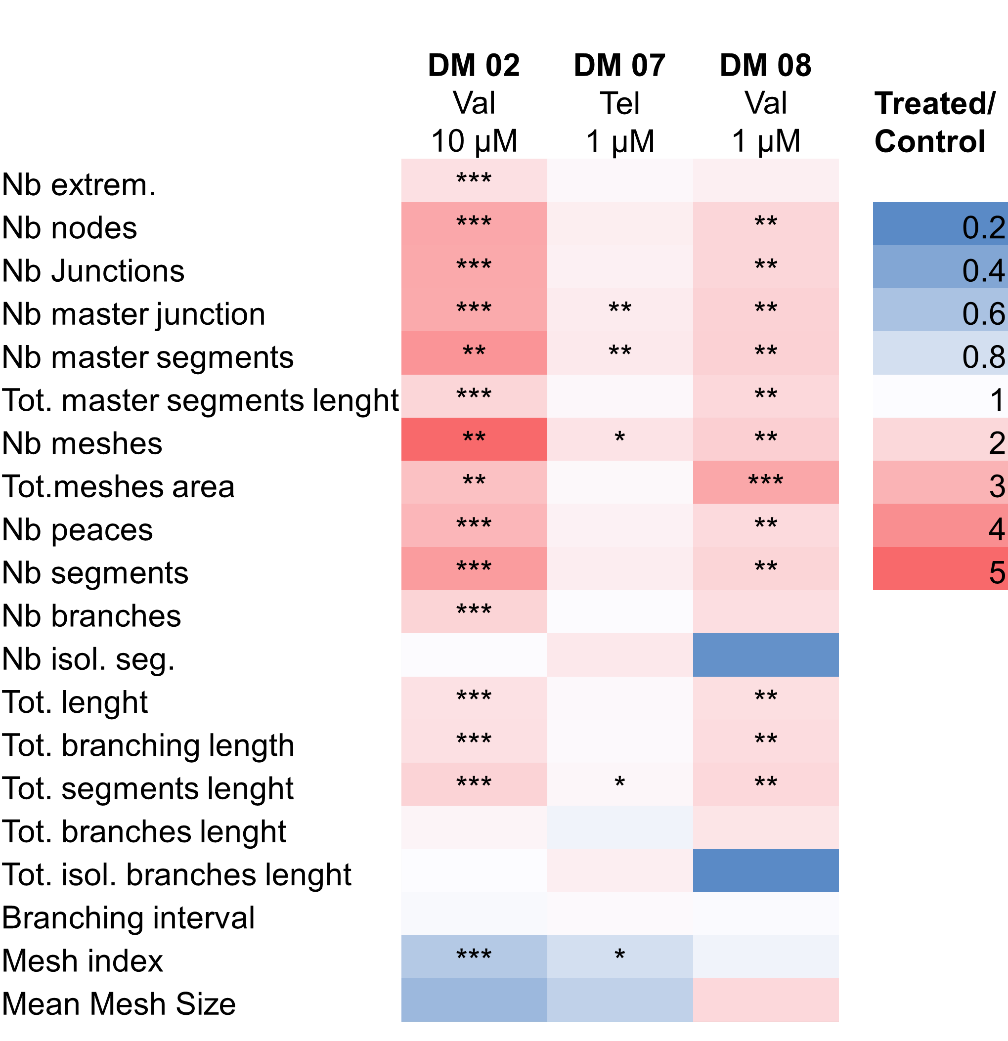
**

**Supplemental Figure S7**. Heatmap of the parameters measured in the tubulogenesis assay after treated with the optimized drug for each line of DiPSC-ECs, relative to the cells without the drug treatment. ***** p<0.05; ** p<0.01; *** p<0.001
