# Supplementary material for: Modeling diabetic endothelial dysfunction with patient‐specific induced pluripotent stem cells
Source: Bioeng Transl Med. 2023 Aug 30;8(6):e10592. doi: 10.1002/btm2.10592 (PMC10658533; doi:10.1002/btm2.10592)
Supplement: Supplementary file 2 — Table S1. DNA fingerprinting STR profiles of PBMCs, iPSCs and iPSC‐ECs from each human subject by Cell Line Genetics (Madison, WI). [file BTM2-8-e10592-s004.docx]

**Supplemental Table S1.** DNA fingerprinting STR profiles of PBMCs, iPSCs and iPSC-ECs from each human subject by Cell Line Genetics (Madison, WI).

| **Healthy** | **AMH 01** | | | | | | **AMH 03** | | | | | | **AMH 04** | | | | | |
| --- | --- | --- | --- | --- | --- | --- | --- | --- | --- | --- | --- | --- | --- | --- | --- | --- | --- | --- |
|  | **PBMCs** | | **iPSCs** | | **iPSC-ECs** | | **PBMCs** | | **iPSCs** | | **iPSC-ECs** | | **PBMCs** | | **iPSCs** | | **iPSC-ECs** | |
| Amelogenin | X |  | X |  | X |  | X |  | X |  | X |  | X | Y | X | Y | X | Y |
| vWA | 16 | 17 | 16 | 17 | 16 | 17 | 15 | 17 | 15 | 17 | 15 | 17 | 14 | 16 | 14 | 16 | 14 | 16 |
| D8S1179 | 11 | 14 | 11 | 14 | 11 | 14 | 12 | 14 | 12 | 14 | 12 | 14 | 10 | 14 | 10 | 14 | 10 | 14 |
| TPOX | 8 | 10 | 8 | 10 | 8 | 10 | 8 | 11 | 8 | 11 | 8 | 11 | 8 | 11 | 8 | 11 | 8 | 11 |
| FGA | 22 |  | 22 |  | 22 |  | 19 | 23 | 19 | 23 | 19 | 23 | 22 | 29 | 22 | 29 | 22 | 29 |
| D3S1358 | 15 | 17 | 15 | 17 | 15 | 17 | 15 | 17 | 15 | 17 | 15 | 17 | 16 | 18 | 16 | 18 | 16 | 18 |
| THO1 | 7 | 9 | 7 | 9 | 7 | 9 | 6 | 9 | 6 | 9 | 6 | 9 | 7 |  | 7 |  | 7 |  |
| D21S11 | 29 | 30 | 29 | 30 | 29 | 30 | 29 | 32.2 | 29 | 32.2 | 29 | 32.2 | 28 |  | 28 |  | 28 |  |
| D18S51 | 14 |  | 14 |  | 14 |  | 12 | 20 | 12 | 20 | 12 | 20 | 16 | 18 | 16 | 18 | 16 | 18 |
| Penta E | 7 | 9 | 7 | 9 | 7 | 9 | 7 | 12 | 7 | 12 | 7 | 12 | 8 | 13 | 8 | 13 | 8 | 13 |
| D5S818 | 13 |  | 13 |  | 13 |  | 11 | 12 | 11 | 12 | 11 | 12 | 11 | 13 | 11 | 13 | 11 | 13 |
| D13S317 | 12 | 13 | 12 | 13 | 12 | 13 | 11 | 12 | 11 | 12 | 11 | 12 | 11 | 13 | 11 | 13 | 11 | 13 |
| D7S820 | 9 | 11 | 9 | 11 | 9 | 11 | 10 | 12 | 10 | 12 | 10 | 12 | 9 | 10 | 9 | 10 | 9 | 10 |
| D16S539 | 14 |  | 14 |  | 14 |  | 11 | 12 | 11 | 12 | 11 | 12 | 9 | 11 | 9 | 11 | 9 | 11 |
| CSF1PO | 12 |  | 12 |  | 12 |  | 10 | 11 | 10 | 11 | 10 | 11 | 10 | 11 | 10 | 11 | 10 | 11 |
| Penta D | 10 |  | 10 |  | 10 |  | 13 |  | 13 |  | 13 |  | 6 | 9 | 6 | 9 | 6 | 9 |
|  | | | | | | | | | | | | | | | | | | |
| **Diabetic** | **DM 02** | | | | | | **DM 07** | | | | | | **DM 08** | | | | | |
|  | **PBMCs** | | **iPSCs** | | **iPSC-ECs** | | **PBMCs** | | **iPSCs** | | **iPSC-ECs** | | **PBMCs** | | **iPSCs** | | **iPSC-ECs** | |
| Amelogenin | X |  | X |  | X |  | X | Y | X | Y | X | Y | X | Y | X | Y | X | Y |
| vWA | 17 |  | 17 |  | 17 |  | 18 | 19 | 18 | 19 | 18 | 19 | 15 |  | 15 |  | 15 |  |
| D8S1179 | 13 | 14 | 13 | 14 | 13 | 14 | 9 | 10 | 9 | 10 | 9 | 10 | 13 | 15 | 13 | 15 | 13 | 15 |
| TPOX | 8 |  | 8 |  | 8 |  | 8 | 9 | 8 | 9 | 8 | 9 | 9 | 10 | 9 | 10 | 9 | 10 |
| FGA | 22 |  | 22 |  | 22 |  | 21 | 22 | 21 | 22 | 21 | 22 | 19 | 23 | 19 | 23 | 19 | 23 |
| D3S1358 | 17 | 18 | 17 | 18 | 17 | 18 | 16 | 17 | 16 | 17 | 16 | 17 | 16 | 18 | 16 | 18 | 16 | 18 |
| THO1 | 6 | 8 | 6 | 8 | 6 | 8 | 6 | 9.3 | 6 | 9.3 | 6 | 9.3 | 6 | 9.3 | 6 | 9.3 | 6 | 9.3 |
| D21S11 | 32.2 |  | 32.2 |  | 32.2 |  | 27 | 28 | 27 | 28 | 27 | 28 | 28 |  | 28 |  | 28 |  |
| D18S51 | 15 | 17 | 15 | 17 | 15 | 17 | 11 | 14 | 11 | 14 | 11 | 14 | 17 | 19 | 17 | 19 | 17 | 19 |
| Penta E | 12 | 13 | 12 | 13 | 12 | 13 | 7 | 10 | 7 | 10 | 7 | 10 | 7 | 13 | 7 | 13 | 7 | 13 |
| D5S818 | 10 | 11 | 10 | 11 | 10 | 11 | 12 | 13 | 12 | 13 | 12 | 13 | 12 | 13 | 12 | 13 | 12 | 13 |
| D13S317 | 10 | 12 | 10 | 12 | 10 | 12 | 10 | 12 | 10 | 12 | 10 | 12 | 11 | 13 | 11 | 13 | 11 | 13 |
| D7S820 | 10 | 11 | 10 | 11 | 10 | 11 | 11 | 13 | 11 | 13 | 11 | 13 | 8 | 11 | 8 | 11 | 8 | 11 |
| D16S539 | 12 | 13 | 12 | 13 | 12 | 13 | 11 | 13 | 11 | 13 | 11 | 13 | 12 |  | 12 |  | 12 |  |
| CSF1PO | 12 | 13 | 12 | 13 | 12 | 13 | 10 | 12 | 10 | 12 | 10 | 12 | 10 | 11 | 10 | 11 | 10 | 11 |
| Penta D | 10 | 11 | 10 | 11 | 10 | 11 | 9 | 13 | 9 | 13 | 9 | 13 | 2.2 | 13 | 2.2 | 13 | 2.2 | 13 |
