# Supplementary material for: Modeling diabetic endothelial dysfunction with patient‐specific induced pluripotent stem cells
Source: Bioeng Transl Med. 2023 Aug 30;8(6):e10592. doi: 10.1002/btm2.10592 (PMC10658533; doi:10.1002/btm2.10592)
Supplement: Supplementary file 6 — Table S5. List of antibodies used in this study for immunofluorescence imaging (i), flow cytometry (f), western blotting (w), and cell‐based ELISA (e). [file BTM2-8-e10592-s006.docx]

**Supplemental Table S5.** List of antibodies used in this study for immunofluorescence imaging (i), flow cytometry (f), western blotting (w), and cell-based ELISA (e).

| **Target Antigen (application)** | **Vendor** | **Catalog #** | **Host Isotype** | **Conjugate** | **Dilution** |
| --- | --- | --- | --- | --- | --- |
| OCT-4 (i) | Thermo-Fisher | 701756 | Rabbit IgG |  | 1:500 |
| SSEA4 (i) | Thermo-Fisher | 414000 | Mouse IgG |  | 1:500 |
| TRA-1-60 (i) | Thermo-Fisher | MA1-023 | Mouse IgM |  | 1:100 |
| Alkaline Phosphatase (w) | Abcam | ab203106 | Rabbit IgG |  | 1:200 |
| Alpha Fetoprotein (i) | Thermo-Fisher | PA5-16658 | Rabbit IgG |  | 1:100 |
| Desmin (i) | Thermo-Fisher | MA1-06401 | Mouse IgG |  | 1:100 |
| Beta-3 Tubulin (i) | Thermo-Fisher | MA1-118 | Mouse IgG |  | 1:100 |
| Beta Actin (w) | Thermo-Fisher | AM4302 | Mouse IgG |  | 1:200 |
| VE-Cadherin (f) | Thermo-Fisher | 12-1449-82 | Mouse IgG | PE | 1:200 |
| PECAM-1 (f) | Thermo-Fisher | 11-0319-42 | Mouse IgG | FITC | 1:200 |
| PECAM-1 (i) | Sigma-Aldrich | P-8590 | Mouse IgG |  | 1:250 |
| VE-Cadherin (i) | Sigma-Aldrich | V1514 | Rabbit IgG |  | 1:250 |
| VE-Cadherin (w) | Sigma-Aldrich | V1514 | Rabbit IgG |  | 1:500 |
| FLK-1 (i) | Santa Cruz | sc-6251 | Mouse IgG |  | 1:250 |
| CD41(i) | Abcam | ab11024 | Mouse IgG |  | 1:100 |
| ICAM-1 (i) | Thermo-Fisher | MA5-13021 | Mouse IgG |  | 1:100 |
| ICAM-1 (e) | Thermo-Fisher | MA5-13021 | Mouse IgG |  | 1:750 |
| ICAM-1 (w) | Thermo-Fisher | MA5407 | Mouse IgG |  | 1:250 |
| VCAM-1 (i) | Thermo-Fisher | PA5-112066 | Rabbit IgG |  | 1:300 |
| VCAM-1 (e) | Thermo-Fisher | PA5-112066 | Rabbit IgG |  | 1:750 |
| VCAM-1 (w) | Thermo-Fisher | PA5-112066 | Rabbit IgG |  | 1:500 |
| P-Selectin (i) | Thermo-Fisher | 701257 | Rabbit IgG |  | 1:100 |
| P-Selectin (e) | Thermo-Fisher | 701257 | Rabbit IgG |  | 1:200 |
| AGTR1 | Thermo-Fisher | AAR-011 | Rabbit IgG |  | 1:500 |
| Mouse IgM (i) | Thermo-Fisher | A-21042 | Goat IgG | Alexa Fluor™ 488 | 1:1000 |
| Mouse IgG (i) | Thermo-Fisher | A-11001 | Goat IgG | Alexa Fluor™ 488 | 1:1000 |
| Mouse IgG (i) | Thermo-Fisher | A32742 | Goat IgG | Alexa Fluor™ Plus 594 | 1:1000 |
| Rabbit IgG (i) | Thermo-Fisher | A32754 | Donkey IgG | Alexa Fluor™ Plus 594 | 1:1000 |
| Mouse IgG (e) | Sigma-Aldrich | G21040 | Goat IgG | HRP | 1:2000 |
| Mouse IgG (w) | Sigma-Aldrich | G21040 | Goat IgG | HRP | 1:10,000 |
| Rabbit IgG (e) | Thermo-Fisher | 31460 | Goat IgG | HRP | 1:3000 |
| Rabbit IgG (w) | Thermo-Fisher | 31460 | Goat IgG | HRP | 1:10,000 |
